# Tuning local chemistry of P2 layered-oxide cathode for high energy and long cycles of sodium-ion battery

**DOI:** 10.1038/s41467-021-22523-3

**Published:** 2021-04-15

**Authors:** Chenchen Wang, Luojia Liu, Shuo Zhao, Yanchen Liu, Yubo Yang, Haijun Yu, Suwon Lee, Gi-Hyeok Lee, Yong-Mook Kang, Rong Liu, Fujun Li, Jun Chen

**Affiliations:** 1 Key Laboratory of Advanced Energy Materials Chemistry (Ministry of Education), Renewable Energy Conversion and Storage Center (RECAST), College of Chemistry, Nankai University, Tianjin, China; 2 College of Materials Science and Engineering, Beijing University of Technology, Beijing, China; 3 Department of Materials Science and Engineering, Korea University, Seoul, Republic of Korea; 4 Department of Materials Science and Engineering, Dongguk University, Seoul, Republic of Korea; 5 Advanced Light Source, Lawrence Berkeley National Laboratory, Berkeley, CA USA; 6 Secondary Ion Mass Spectrometry Facility, Western Sydney University, Locked 17 Bag 1797, Penrith, NSW Australia

**Keywords:** Batteries, Electronic devices, Batteries

## Abstract

Layered transition-metal oxides have attracted intensive interest for cathode materials of sodium-ion batteries. However, they are hindered by the limited capacity and inferior phase transition due to the gliding of transition-metal layers upon Na^+^ extraction and insertion in the cathode materials. Here, we report that the large-sized K^+^ is riveted in the prismatic Na^+^ sites of P2-Na_0.612_K_0.056_MnO_2_ to enable more thermodynamically favorable Na^+^ vacancies. The Mn-O bonds are reinforced to reduce phase transition during charge and discharge. 0.901 Na^+^ per formula are reversibly extracted and inserted, in which only the two-phase transition of P2 ↔ P’2 occurs at low voltages. It exhibits the highest specific capacity of 240.5 mAh g^−1^ and energy density of 654 Wh kg^−1^ based on the redox of Mn^3+^/Mn^4+^, and a capacity retention of 98.2% after 100 cycles. This investigation will shed lights on the tuneable chemical environments of transition-metal oxides for advanced cathode materials and promote the development of sodium-ion batteries.

## Abstract (web summary)

High-capacity and structural stable cathode materials are challenges for sodium-ion batteries. Here, the authors report a layered P2-Na_0.612_K_0.056_MnO_2_ with large-sized K^+^ riveted in the Na-layers to enable 0.9 Na^+^ (de)insertion with a reversible phase transition of P2-P’2.

## Introduction

Sodium-ion batteries (SIBs) have been considered as one promising next-generation battery system for large-scale energy storage and smart electric grid, due to the low cost and wide distribution of Na resources^1–3^. The battery performance and cost are mainly governed by the applied cathode materials^4–7^. A wide range of host materials, such as transition-metal oxides, phosphates, Prussian blue analogs, and so on, have been investigated for reversible (de)intercalation of Na^+^. However, the intrinsic large ionic radius of 102 pm and molar weight of Na^+^ over its Li^+^ counterpart impose penalties on the specific capacity or the structure stability of cathode materials, which limit the deployment of SIBs^8–12^. It is rational to design and synthesize cathode materials with more available sites for Na^+^ storage and robust crystal structure for Na^+^ (de)intercalation, ensuring their large specific capacities and long cycle life.

Layered transition-metal oxides with earth-abundant elements have been actively studied as host materials for Na^+^ storage^13,14^. Sodium manganese oxide with a P2 phase (P2-Na_*x*_MnO_2_) is of significant importance for SIBs, which is consisted of two MnO_6_-octahedra slabs and sandwiched Na layers in each unit cell with the ABBA oxygen packing frameworks^15,16^. It usually delivers a capacity of ~160 mAh g^−1^, corresponding to insertion of 0.60 Na^+^ per formula for P2-Na_*x*_MnO_2_^17,18^. The limited movable Na^+^ and phase transitions occurring upon its extraction and reinsertion are the major challenges. The Na^+^ extraction to *x* < 0.35 in P2-Na_*x*_MnO_2_ results in the irreversible P2 → OP4 phase transition, due to gliding of the MnO_2_-slabs, and consequently decaying capacities in cycles^19,20^. Fe-doping in the MnO_2_-slabs of Na_*x*_Fe_0.5_Mn_0.5_O_2_ is beneficial for a high capacity of 200 mAh g^−1^ but with 35% capacity loss after 30 cycles, which is originated from the P2 → O2/OP4 phase transition^21^. The capacity can also be promoted to 185 mAh g^−1^ by co-doping Co and Ni for P2-Na_0.7_Mn_0.6_Ni_0.3_Co_0.1_O_2_ (Yoshida et al.^22^). Partial Mg substitution for Mn in P2-Na_*x*_MnO_2_ delays the formation of OP4 phase, leading to smooth electrochemical profiles and extended cycling performance with a capacity of 106 mAh g^−1^ (Clément et al.^19^). The substitution of Li or Al in Na_*x*_MnO_2_ can suppress the Jahn-Teller distortion and enhance its phase stability^23,24^. It is clear that finely tuning the chemical environments of the MnO_6_-octahedra with suitable dopants in the place of Mn allows for either more accessible Na^+^ sites for large capacities with limited cycles or minimized slab gliding for long cycles with moderate capacities. However, solutions to such paradox are challenging and highly desirable for the development of SIBs^25^.

Here, we show that the large-sized K^+^ of 138 pm is riveted in the edge-sharing sites of Na^+^ in P2-Na_0.612_K_0.056_MnO_2_, which leads to the small interslab distance of 0.56 nm in Na_0.612_K_0.056_MnO_2_ and results in high diffusion barrier of K^+^ and hence its immobility. The Na-O bond strength and Na^+^ vacancy formation energy are reduced to favor more extractable and insertional Na^+^. The reinforced MnO_6_-octahedra slabs are beneficial to the structure stability of P2-Na_0.612_K_0.056_MnO_2_. In situ X-ray diffraction (XRD) indicates that there is no inferior phase transition at the high charge voltages during cycles. It turns out to exhibit a high specific capacity of 240.5 mAh  g^−1^ with Mn^3+^/Mn^4+^ as the redox couple and the highest energy density of 654 Wh kg^−1^ with good cycle stability and rate capability.

## Results and discussion

### Characterization and structure analysis

**Fig. 1:**
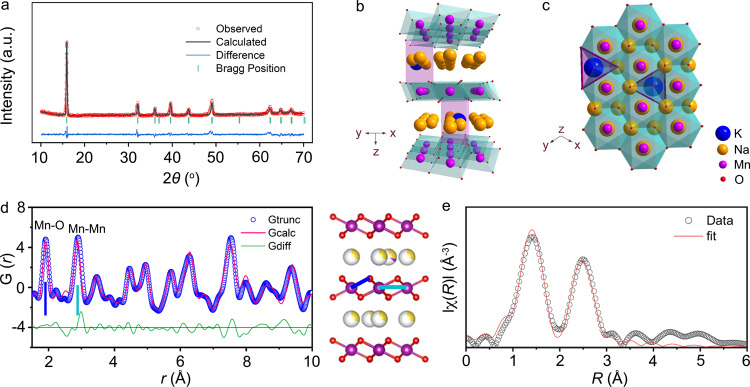
Crystal structure of Na_0.612_K_0.056_MnO_2_. **a** XRD pattern and Rietveld refinement. Typical layered structure of Na_0.612_K_0.056_MnO_2_ viewed along the *y* axis (**b**) and the *z* axis (**c**) with K^+^ located at the Na_e_ sites. **d** PDF pattern and structure of Na_0.612_K_0.056_MnO_2_. The representative peaks correspond to the bond length of Mn–O (blue line) and Mn–Mn (pale blue line) as labeled. **e** Fitting of Mn K-edge FT-EXAFS spectra of Na_0.612_K_0.056_MnO_2_.

Na_0.67−*x*_K_*x*_MnO_2_ (0 ≤ *x* ≤ 0.2) was synthesized via a solid-state reaction at 900 °C in air, however, the pure P2 phase was only obtained with *x* ≤ 0.1, as confirmed by the XRD patterns in Fig. 1a and Supplementary Figs. 1 and 2. The optimized composition was found with nominal *x* = 0.1. Its exact composition was determined to P2-Na_0.612_K_0.056_MnO_2_ by inductively coupled plasma-optical emission spectrometry (ICP-OES) in Supplementary Table 1. The oxygen non-stoichiometry in the as-synthesized P2-Na_0.612_K_0.056_MnO_2-δ_ was determined by iodine stoichiometry titration. As listed in Supplementary Table 2, it shows the atomic ratio of oxygen is 1.985, which is mainly attributed to oxygen vacancies on its surface^26^. The XRD pattern and Rietveld refinement of Na_0.612_K_0.056_MnO_2_ and Na_0.706_MnO_2_ are displayed in Fig. 1a and Supplementary Fig. 3, and all the diffraction peaks are assigned to a hexagonal lattice with a space group of *P6*_*3*_*/mmc*. The refined structure of Na_0.612_K_0.056_MnO_2_ is depicted along the *y* and *z* axes in Fig. 1b, c, respectively. There exist two types of trigonal prismatic sites for Na^+^, which are sharing faces (Na_f_) and edges (Na_e_) with the MnO_6_-octahedra in the two adjacent slabs, respectively, as depicted in Fig. 1c and Supplementary Fig. 4. The two adjacent Na sites are not accessible at the same time, because of their large electrostatic repulsion. Rietveld refinement indicates that K^+^ is located at the Na_e_ site (Supplementary Tables 3 and 4), which is mainly ascribed to the much higher stability of the Na_e_ sites^27,28^. Moreover, the interlayer space (*d*_(Na-O-Na)_) is enlarged to 3.58 Å in Na_0.612_K_0.056_MnO_2_ (3.42 Å in Na_0.706_MnO_2_) due to the larger ionic radii for K^+^ versus Na^+^. On the contrary, the *d*_MnO2_ is reduced to 2.02 Å in Na_0.612_K_0.056_MnO_2_ (2.28 Å in Na_0.706_MnO_2_), which is resulted from the enhanced MnO_2_-slabs. Thus, the distances of adjacent MnO_2_-slabs (*d*-spacing) are reduced in K-doped compounds even with larger-sized K^+^ in the Na layers.

Synchrotron X-ray pair distribution function (PDF) analysis was performed to study the local structure of Na_0.612_K_0.056_MnO_2_ in Fig. 1d. The P2-type layered structure is consistent with XRD refinement, as depicted in Supplementary Table 5. The Mn–O and Mn–Mn bond lengths are 1.890 and 2.891 Å, respectively, which are shorter than those in Na_0.706_MnO_2_ (1.897 and 2.901) in Supplementary Fig. 5 and Table 6. This suggests the strengthened chemical bonds and reinforced interaction of MnO_6_-octahedra slabs. X-ray adsorption near-edge structure (XANES) spectra in Supplementary Fig. 6a imply that Mn has the approximately same average oxidation states in Na_0.612_K_0.056_MnO_2_ and Na_0.706_MnO_2_. The fitted Mn K-edge Fourier-transformed extended X-ray adsorption fine structure (FT-EXAFS) spectrum of Na_0.612_K_0.056_MnO_2_ is shown in Fig. 1e, as well as Na_0.706_MnO_2_ in Supplementary Fig. 6b. As listed in Supplementary Tables 7 and 8, it shows shortened bond lengths of Mn–O (1.889 Å, 1.895 Å for Na_0.706_MnO_2_) and Mn–Mn (2.889 Å, 2.897 Å for Na_0.706_MnO_2_). This is in good agreement with the PDF results and the *d*-spacings of 0.56 and 0.57 nm between the (002) facets in Na_0.612_K_0.056_MnO_2_ and Na_0.706_MnO_2_, respectively, as observed by high-resolution transmission electron microscopy (HRTEM) in Supplementary Fig. 7.

**Fig. 2:**
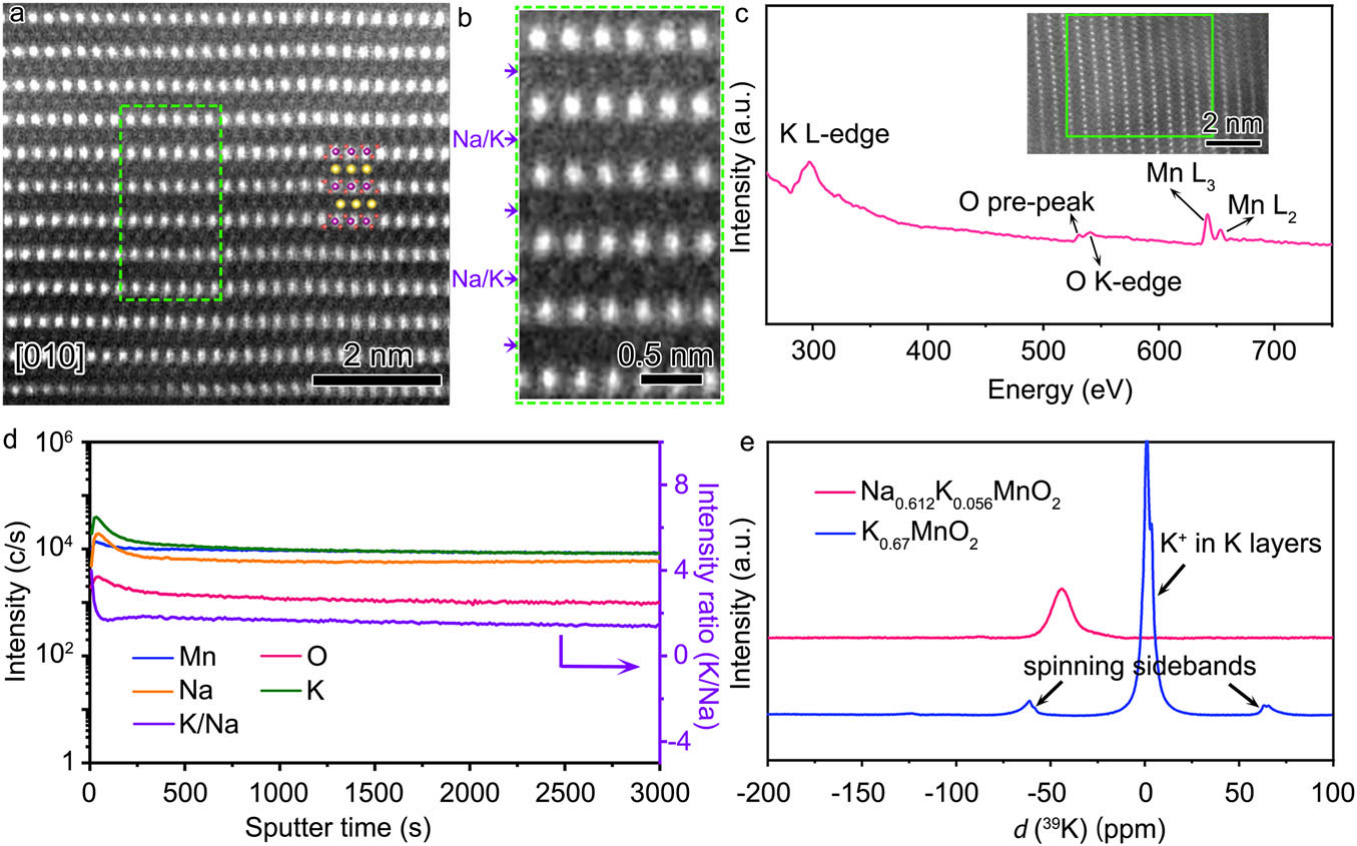
Atomic structure and elemental distribution of Na_0.612_K_0.056_MnO_2_. **a** STEM-HAADF image along the direction of [010] of Na_0.612_K_0.056_MnO_2_. **b** Magnified images of the indicated squares in **a**. **c** EELS spectrum of the selected area in the inset image. **d** SIMS elemental profiles at different sputtering times. **e** Solid-state ^39^K NMR spectra of Na_0.612_K_0.056_MnO_2_ and K_0.67_MnO_2_.

Scanning transmission electron microscopy (STEM) equipped with the detectors of high-angle annular dark field (HAADF) and annular bright field (ABF) are employed to study the atomic structure of Na_0.612_K_0.056_MnO_2_ in Fig. 2a, b and Supplementary Fig. 8, which is 1–10 μm in size in Supplementary Fig. 9a. The bright dots in the STEM-HAADF images and the dark dots in STEM-ABF image are assigned to the atomic columns of Mn, between which are the atomic columns of Na or K. The STEM-HAADF and SEM images in Supplementary Figs. 9b and 10 indicate that Na_0.706_MnO_2_ has similar morphology and layered structure with Na_0.612_K_0.056_MnO_2_. Electron energy loss spectroscopy (EELS) in Fig. 2c and Supplementary Fig. 11 were collected in the selected areas as indicated, in which the peaks of K L-edge at 297 eV indicate the presence of K in the Na layers in Na_0.612_K_0.056_MnO_2_.

Dynamic secondary ion mass spectroscopy (SIMS) is performed to analyze the distribution of elements from the surface to the bulk of Na_0.612_K_0.056_MnO_2_ in Fig. 2d, as well as their elemental mappings in Supplementary Figs. 12 and 13. It is shown that the contents of K and Na are increased along with a slight decrease of Mn in the initial 50 s of sputtering, and then they are gradually dropped and become constant. The K profile shows a slight aggregation trend on the surface in the initial 50 s of sputtering, and then the intensity ratio of K and Na is gradually decreased to a stable value, indicating uniform distribution of K and Na in the bulk of Na_0.612_K_0.056_MnO_2_. Similar trends for Na, Mn, and O are observed in the SIMS spectra of Na_0.706_MnO_2_ in Supplementary Fig. 14. Solid-state ^39^K nuclear magnetic resonance (NMR) spectrum of Na_0.612_K_0.056_MnO_2_ with K_0.67_MnO_2_ as a reference is shown in Fig. 2e. The peak at ~0 ppm is assigned to K^+^ located in the K-layers in the crystal structure of K_0.67_MnO_2_. The other two weak peaks at 70 and −70 ppm appear symmetrically and are assigned to the spinning sidebands due to the rotation of sample tube^29^. A negative chemical shift is observed for Na_0.612_K_0.056_MnO_2_, which is attributed to the smaller electronic shielding effect of Na and O in Na_0.612_K_0.056_MnO_2_ than K and O in K_0.67_MnO_2_, and a larger *d*-spacing of 0.64 nm in K_0.67_MnO_2_ than in P2-Na_0.612_K_0.056_MnO_2_ with the similar layered structure, as shown in Supplementary Fig. 15 and Table 9_._ These imply that K^+^ exists only in the Na layer, not in the MnO_2_-layer, and has a uniform distribution in the bulk particles of Na_0.612_K_0.056_MnO_2_.

### Reaction mechanism

**Fig. 3:**
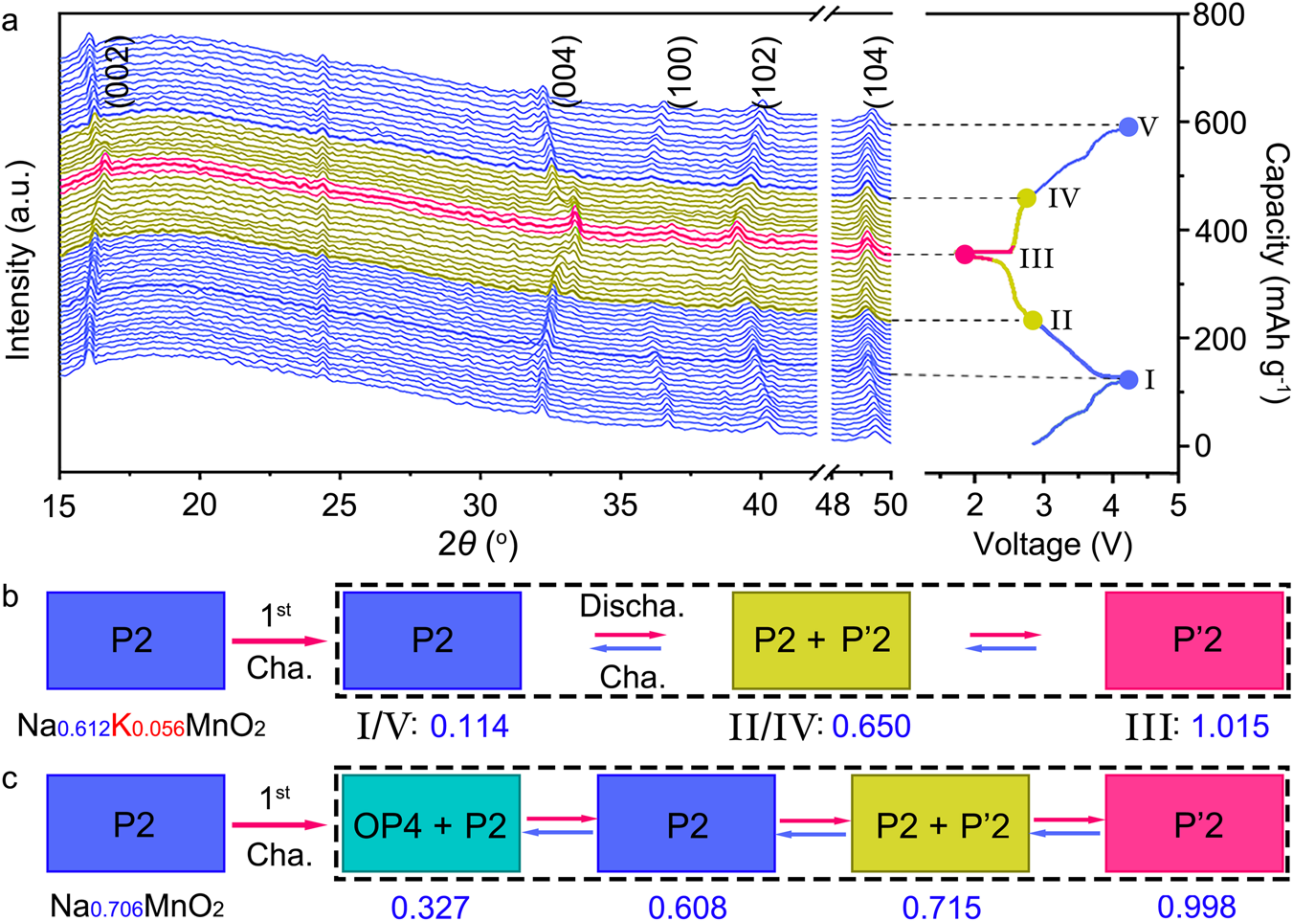
Structure evolution during charge and discharge. **a** In situ XRD patterns corresponding to the charge and discharge curves of Na_0.612_K_0.056_MnO_2_. Schematic illustration of phase transitions along with the content change of Na^+^ in Na_0.612_K_0.056_MnO_2_ (**b**) and Na_0706_MnO_2_ (**c**) in cycles of charge and discharge.

The structure evolution of Na_0.612_K_0.056_MnO_2_ upon Na^+^ extraction and intercalation is monitored by in situ XRD in a home-made battery within a voltage window of 1.8–4.3 V. As shown in Fig. 3a, each XRD pattern is obtained during the battery operation and corresponding to the state as indicated in the charge and discharge curves in the right-hand panel. The pristine Na_0.612_K_0.056_MnO_2_ is identified by the characteristic diffraction peaks of the (002), (004), (100), (102), and (104) planes in Fig. 3a. The P2 phase of Na_0.612_K_0.056_MnO_2_ is sustained in the first charge process without phase transition, and 0.498 Na^+^ per formula is extracted to give the fully charged state (I) of Na_0.114_K_0.056_MnO_2_, which agrees well with the ICP-OES in Supplementary Table 1. In the following discharge process, the P2 phase of the host is kept until 0.536 Na^+^ is inserted for Na_0.65_K_0.056_MnO_2_ (II). Due to the slight distortion of the MnO_6_-octahedra slabs, the P’2 phase starts to appear with the co-existence of P2 phase, and becomes dominant in the fully discharged state (III) of Na_1.015_K_0.056_MnO_2_. In the reverse charge process, the P’2 phase is gradually transformed to the P2 phase (IV), and then it is sustained at high charge voltages till 4.3 V (V). It involves the reversible two-phase transition of P2 ↔ P′2 with total insertion or extraction of 0.901 Na^+^ in the cycles of discharge and charge, as schematically illustrated in Fig. 3b. In contrast, P2-Na_0.706_MnO_2_ involves large gliding of the MnO_6_-octahedra slabs for the phase transition of P2 → OP4 upon extraction of 0.455 Na^+^ at high charge voltages; it then converts from the OP4 phase into P2 and P′2 during discharge with total insertion or extraction of 0.671 Na^+^ in cycles, as shown in the schematic phase transitions of P2-Na_0.706_MnO_2_ in Fig. 3c and in situ XRD patterns in Supplementary Fig. 17. Rietveld refinements of the fully charged and discharged Na_0.612_K_0.056_MnO_2_ and P2-Na_0.706_MnO_2_ (Supplementary Figs. 18–21 and Supplementary Tables 10–13) show drastic changes in the crystal structure and lattice parameters of P2-Na_0.706_MnO_2_. It was found that the volume variation of Na_*x*_K_0.065_MnO_2_ is 7.8%, which is significantly smaller than 11.2% in Na_*x*_MnO_2_, and favors the structural stability during cycling. It reveals that more Na^+^ (0.901 vs. 0.671 Na^+^) can be extracted at the high charge voltages and re-inserted from/into P2-Na_0.612_K_0.056_MnO_2_ than P2-Na_0.706_MnO_2_, due to the reduced Na-O bond strength and reinforced MnO_6_-octahedra slabs for suppression of the inferior OP4 phase.

**Fig. 4:**
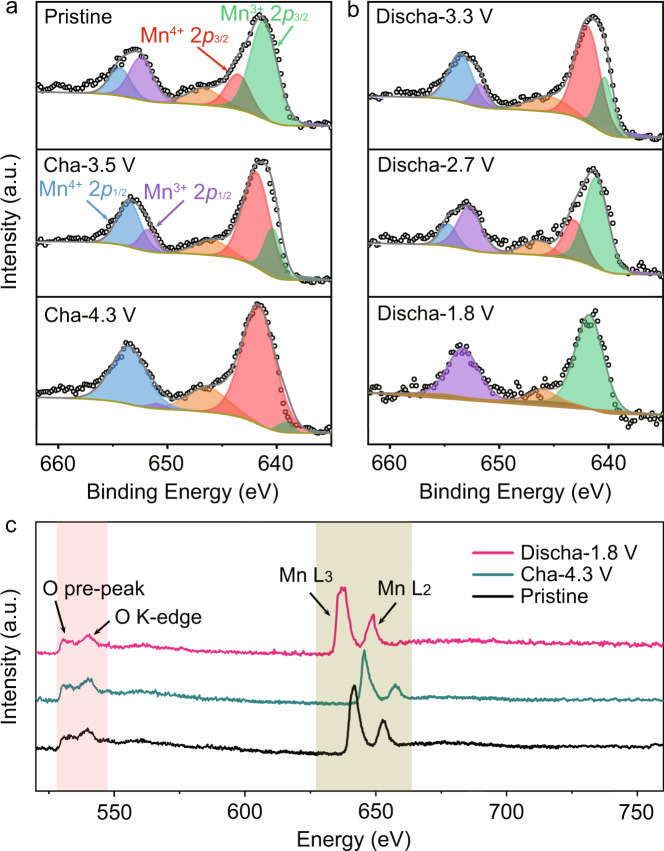
Valence evolution during charge and discharge of Na_0.612_K_0.056_MnO_2_. XPS Mn 2*p* spectra at the charge (**a**) and discharge (**b**) states. **c** EELS spectra at different states of Na_0.612_K_0.056_MnO_2_.

The redox of Na_0.612_K_0.056_MnO_2_ is further unveiled by X-ray photoelectron spectroscopy (XPS) and EELS. The XPS Mn 2*p* spectrum of Na_0.612_K_0.056_MnO_2_ in Fig. 4a shows the splitting Mn 2*p*_3/2_ and Mn 2*p*_1/2_ at 640.8/642.2 and 652.4/654.9 eV, respectively, suggesting the co-existence of Mn^3+^ and Mn^4+^ with a ratio of 2:1. Upon charging, the intensity of Mn^4+^ peaks increases with decrease of the intensity of Mn^3+^ peaks, indicating oxidation of Mn^3+^ to Mn^4+^ in Fig. 4a. The remaining Mn^3+^ is 0.08 per formula at the charge finish of 4.3 V, which is consistent with the phase transition in Fig. 3b. The reduction of Mn^4+^ to Mn^3+^ in the following discharge process is evidenced by the gradual intensity increase of Mn^3+^ peaks till the disappearance of Mn^4+^ peaks, as shown in Fig. 4b. The peaks at 646.5 eV in Fig. 4a, b are mainly attributed to Augers of C–F and Na–F^30^. In addition, the EELS spectra of the charged, discharged, and pristine states of Na_0.612_K_0.056_MnO_2_ in Fig. 4c further confirm the redox of Mn^3+^/Mn^4+^ with the reversible shifts of the two peaks of Mn-L_2_ and Mn-L_3_. It is notable that there is no peak shift or broadening in both of the O pre-peak and K-edge, which suggests no valence change of O^2-^. Therefore, the redox of Mn^3+^/Mn^4+^ is responsible for the charge compensation in the extraction and reinsertion of Na^+^ from/into Na_0.612_K_0.056_MnO_2_ in the discharge and charge processes. Also, no oxygen evolution was detected by in situ differential electrochemical mass spectrometry (DEMS) in the charge process (Supplementary Fig. 22).

**Fig. 5:**
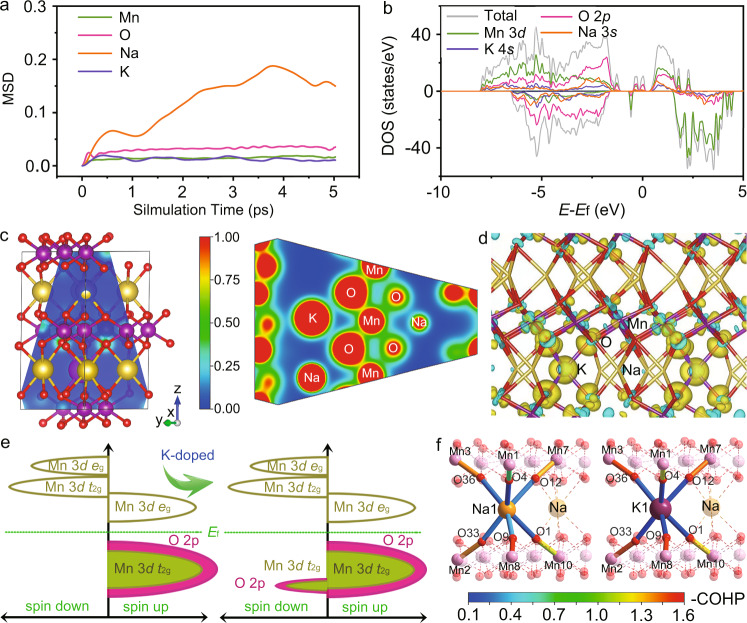
Simulated atomic and electronic structures. **a** MSD of Mn, K, Na, and O as functions of time in Na_0.500_K_0.055_MnO_2_. **b** Total density of states (tDOS) of Na_0.220_K_0.055_MnO_2_ and partial density of states (pDOS) of Na 3 *s*, K 4 *s*, O 2*p* and Mn 3*d* orbitals, as labeled by yellow, purple, red and green, respectively. The Fermi energy (*E*_f_) is set to 0 eV, and the pDOS of Na 3 *s*, K 4 *s* are enlarged by 100 times. **c** Partial charge density distribution along the blue plane in the left panel for Na_0.500_K_0.055_MnO_2_ (visualized by Visualization for Electronic and Structure Analysis (VESTA)). The red regions represent high charge density, and the blue regions indicate low charge density. **d** Plot of deformation charge densities of Na_0.500_K_0.055_MnO_2_. The charge accumulation region is rendered in yellow, and the charge depletion is in blue. **e** Schematic pDOS of Na_0.555_MnO_2_ and Na_0.500_K_0.055_MnO_2_. **f** COHP analysis on representative prismatic sites and the adjacent MnO_6_-octahedra sites of Na_0.555_MnO_2_ and Na_0.500_K_0.055_MnO_2_.

Density functional theory (DFT) calculations using Vienna Ab-initio Simulation Package (VASP) are performed to optimize the crystal structures of the pristine and charged cathode materials^31–34^. Because the numbers of K and Na atoms can only be integral in a unit cell, there exists a slight difference between the simulated formula and the obtained composition, which can be reasonably ignored. Figure 5a and Supplementary Fig. 23 exhibit the first-principle molecular dynamics simulations of Na_0.500_K_0.055_MnO_2_ and Na_0.555_MnO_2_, respectively, where the mean square displacements (MSDs) from 0 to 5 ps reflect the diffusion capability of each element. It shows that Na^+^ is more facile to migrate in Na_0.500_K_0.055_MnO_2_, and K^+^ is immobile as riveted between layers. Its migration is energetically unfavorable as displayed in Supplementary Fig. 24, in which its migration energy barrier is as high as ~0.8 eV in contrast with ~0.4 eV for Na^+^ in Na_0.500_K_0.055_MnO_2_ (refs. ^35–37^). Its diffusion coefficient is accordingly five orders of magnitude lower than that of Na^+^ based on Arrhenius equation. This is consistent with ICP-OES and elemental mappings in Supplementary Table 1 and Figs. 25 and 26, in which the content of K remains almost constant, suggesting no K loss in the charge and discharge processes. The optimized Na_0.500_K_0.055_MnO_2_ in Supplementary Fig. 27a shows a pure P2 phase with K^+^ located in the Na layers. It can be sustained in the optimized structure of P2-Na_0.220_K_0.055_MnO_2_ at the fully charged state with similar structure parameters in Supplementary Fig. 27b. This indicates the significant role of K for the stabilization of the crystal structure of Na_0.612_K_0.056_MnO_2_ for extraction and insertion of Na^+^. It is robust to allow for the extraction of 0.498 Na^+^ in the first charge without phase transition, as depicted in Fig. 3.

The electronic structures of Na_0.500_K_0.055_MnO_2_ and Na_0.555_MnO_2_ have been investigated by DFT calculations. As shown in the density of states (DOS) of Na_0.220_K_0.055_MnO_2_ in Fig. 5b, although K only accounts for one fourth of Na in the composition, the DOS contributed by K atoms is similar to that of Na atoms. In addition, the partial charge density distribution in Fig. 5c reflects the local status of electrons. The electron cloud overlap between K and O is stronger than that between Na and O. As seen from the deformation charge densities for Na_0.500_K_0.055_MnO_2_ in Fig. 5d, the electron population at O atoms increases. However, the crystal orbital Hamilton populations (COHP) in Supplementary Fig. 28 indicate that it falls in a K-O antibonding region around the Fermi level, which is different from the bonding area of Na–O in Na_0.555_MnO_2_. Schematic pDOS of Na_0.500_K_0.055_MnO_2_ and Na_0.555_MnO_2_ are shown in Fig. 5e and Supplementary Figs. 29 and 30. The increased spin-down electrons around Fermi energy indicate stronger hybridization interaction between Mn and O in the presence of K^+^, in good agreement with the analysis of FT-EXAFS in Fig. 1d. Also, the Na-O bond strength in Na_0.500_K_0.055_MnO_2_ is found to be reduced by COHP analysis in Supplementary Table 14. As indicated by COHP analysis in Fig. 5f and Supplementary Tables 15–17, the Mn–O bonds are reinforced for both adjacent and distant Mn atoms with a more obvious increase in *xy*-plane by the substitution of K. A slight fluctuation of Mn–O bond strength is found for different valence state of Mn. These lead to more thermodynamically favorable Na^+^ vacancies (Supplementary Table 18), which is the determining factor for Na^+^ extraction and insertion, consistent with MSD in Fig. 5a. Of note, the formation energy for K^+^ siting in the Na_e_ site (0.675 eV) is higher than in the Na_f_ site (−3.500 eV) of Na_0.500_K_0.055_MnO_2_, which indicates the higher thermodynamic stability of the Na_e_ site for K^+^. Furthermore, the reinforced Mn–O bonds play an important role to suppress the phase transition during Na^+^ extraction and insertion and favor the cycle stability.

### Electrochemical performance

**Fig. 6:**
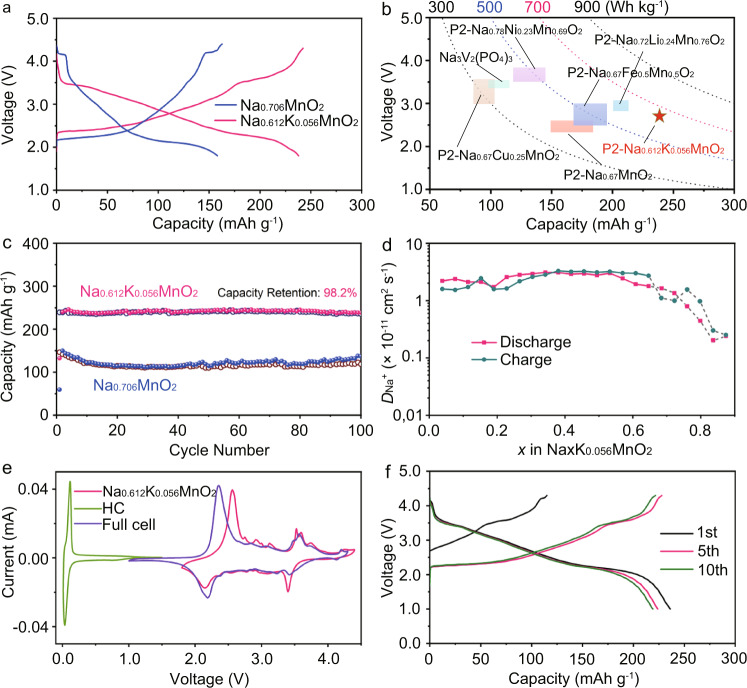
Electrochemical performance. **a** Typical charge/discharge curves of Na_0.612_K_0.056_MnO_2_ and Na_0.706_MnO_2_ at 20 mA g^−1^ in the third cycle. **b** Comparison of energy density and average voltages of Na_0.612_K_0.056_MnO_2_ with typical cathode materials in refs. ^38^^–^^45^. **c** Cycle performance of Na_0.612_K_0.056_MnO_2_ and Na_0.706_MnO_2_ at 50 mA g^−1^. **d** Variation of *D*_Na_ + as functions of x in Na_*x*_K_0.056_MnO_2_ determined by GITT and CV, where the gray dotted area represents the two-phase region. **e** CV curves of Na_0.612_K_0.056_MnO_2_, HC and the full battery of HC//Na_0.612_K_0.056_MnO_2_ at 0.1 mV s^−1^. **f** Typical charge/discharge curves of HC//Na_0.612_K_0.056_MnO_2_ at 50 mA g^−1^.

The electrochemical performance of Na_0.612_K_0.056_MnO_2_ is evaluated against Na metal anodes with the electrolyte of 1.0 M NaPF_6_ in propylene carbonate (PC) and 5 wt.% of fluoroethylene carbonate (FEC) in coin cells. Figure 6a exhibits the typical charge and discharge profiles of Na_0.612_K_0.056_MnO_2_ and Na_0.706_MnO_2_ at 20 mA g^−1^. Na_0.612_K_0.056_MnO_2_ delivers a high initial charge capacity of 125.1 mAh g^−1^, corresponding to 0.498 Na^+^ per formula. In the following cycles, the reversible capacity of Na_0.612_K_0.056_MnO_2_ reaches up to 240.5 mAh g^−1^, which is much higher than that of Na_0.706_MnO_2_ (167.2 mAh g^−1^). This means that 0.901 Na^+^ per formula is inserted and extracted into/from Na_0.612_K_0.056_MnO_2_. It agrees well with the restricted phase transition in Fig. 3. At the same time, Na_0.612_K_0.056_MnO_2_ presents higher discharge voltages than Na_0.706_MnO_2_ does, which is consistent with the cyclic voltammetry (CV) curves in Supplementary Fig. 31. The energy density of Na_0.612_K_0.056_MnO_2_ is as high as 654 Wh kg^−1^, in comparison with the reported cathode materials in Fig. 6b. Of particular, it is even higher than that of Na_0.72_Li_0.24_Mn_0.76_O_2_, which works with both the O redox and Mn^3+^/Mn^4+^ to deliver the highest specific capacity in refs. ^38–45^.

The cycle stability of Na_0.612_K_0.056_MnO_2_ is tested at 50 mA g^−1^, and the selected charge/discharge profiles of the 1st, 5th, 10th, and 100th cycles are presented in Supplementary Fig. 32. Except for the 1st charge, the charge/discharge profiles are almost overlapped. The capacity after 100 cycles is sustained for 236.2 mAh g^−1^, corresponding to capacity retention of 98.2% with only 0.018% loss per cycle in Fig. 6c, which is highly contrasting with the fluctuating capacities of Na_0.706_MnO_2_. This implies that the structure of Na_0.612_K_0.056_MnO_2_ is robust upon extraction and insertion of Na^+^ in cycles, as confirmed by in situ XRD in Fig. 3.

The Na^+^ diffusion coefficients are estimated by Galvanostatic intermittent titration technique (GITT) in Supplementary Figs. 33–37. The obtained diffusion coefficients (*D*_Na_+) for the single-phase regions of both Na_*x*_K_0.056_MnO_2_ and Na_*x*_MnO_2_ are in the range of 10^−12^–10^−10^ cm^2^s^−1^, and they exhibit decreasing trends as the sodiation proceeds near the two-phase regions in Fig. 6d and Supplementary Fig. 38. Because the GITT method cannot be applicable to the two-phase regions, CVs at different scan rates are used to calculate the apparent values of *D*_Na+_ in Na_0.612_K_0.056_MnO_2_ and Na_0.706_MnO_2_, as presented in the gray region in Fig. 6d, Supplementary Fig. 39, and Supplementary Table 19. Na_0.612_K_0.056_MnO_2_ exhibits faster Na^+^ transport with the content change of Na during charge and discharge, though there is K^+^ riveted between the layers. Enhanced rate capability is presented in Na_0.612_K_0.056_MnO_2_ in Supplementary Fig. 40, in which the reversible capacities are kept from 238.8 mAh g^−1^ to 211.6 and 110.2 mAh g^−1^ at the currents from 50 mA g^−1^ to 100 and 2000 mA g^−1^, respectively. The demonstrated good cycle performance and rate capability of Na_0.612_K_0.056_MnO_2_ are attributed to the reinforced structure stability and facile Na^+^ transport.

A full battery with P2-Na_0.612_K_0.056_MnO_2_ cathode and presodiated hard carbon (HC) anode was demonstrated. The performance of HC anode was firstly investigated in half battery in Supplementary Fig. 41a. As displayed in Fig. 6e, a pair of redox peaks appear at 0.036/0.113 V for HC anode, and the obtained peaks of the full battery are similar to those of Na_0.612_K_0.056_MnO_2_, implying reversible electrochemical reactions. The full battery delivers a specific capacity of 230.6 mAh g^−1^ based on the mass of active cathode material at 50 mA g^−1^ in Fig. 6f, corresponding to a high energy density of 314.4 Wh kg^−1^ based on the total mass of active anode and cathode materials. It also exhibits good cycling stability and rate performance in Supplementary Fig. 41. The electrochemical performance can be optimized by selecting high efficient HC and Na compensation method. This implies that P2-Na_0.612_K_0.056_MnO_2_ will be of great potential for the high-performance SIBs.

In summary, a cathode material of P2-Na_0.612_K_0.056_MnO_2_ has been successfully obtained with the K^+^ riveted in the prismatic edge-sharing sites of Na_e_ between the MnO_6_-octahedra slabs. The reinforced MnO_6_-octahedra slabs and reduced Na^+^ vacancy formation energy enable more active Na^+^ sites and favor its facile diffusion. This leads to the highest reversible capacity of 240.5 mAh g^−1^, corresponding to 0.901 Na^+^ per formula, and energy density of 654 Wh kg^−1^ based on the redox of Mn^3+^/Mn^4+^ among the reported transition-metal oxide cathodes for SIBs, and good capacity retention of 98.2% after 100 cycles. The reinforced interaction in the MnO_6_-octahedra slabs restricts the layer gliding and ensures the robust crystal structure of P2-Na_0.612_K_0.056_MnO_2_, in which two-phase transition of P2 ↔ P’2 occurs only at low charge and discharge voltages, favoring the structure stability and cycle performance. This strategy to finely tune the local chemistry for high energy density and long cycle life will shed lights into the design of electrode materials for SIBs and beyond.

## Methods

### Materials synthesis

P2-Na_0.67-*x*_K_*x*_MnO_2_ was synthesized via a solid-state reaction. For the synthesis of Na_0.612_K_0.056_MnO_2_, NaCH_3_COOH (99%, Alfa, 12.54 mmol, 10% in excess), K_2_CO_3_ (99%, Alfa, 2.1 mmol, 5% in excess), and MnO_2_ (99%, Alfa, 20 mmol) were mixed in a planetary ball mill (QM-3SP2) at 400 rpm for 12 h in the presence of a small amount of acetone. The as-synthesized precursors were dried at 80 °C for 12 h, and then pressed into a pallet under 20 MPa. It was heated at 500 °C for 2 h and 900 °C for 10 h in air at 5 °C/min. Then, it was cooled down to 200 °C at 5 °C/min and naturally to room temperature, and stored in an argon-filled glovebox. Similar processes were applicable to the synthesis of Na_0.706_MnO_2_ except the addition of K_2_CO_3_.

### Electrochemical measurements

Coin cells (CR2032) were assembled in an argon-filled glovebox for all the electrochemical tests. The working electrode was prepared by coating the mixture of active material (80 wt.%), Super P (10 wt.%), and polytetrafluoroethylene (10 wt.%) in *N*-methyl-2-pyrrolidone onto an Al foil. The loading mass of active material in each electrode pellet of 12.0 mm in diameter was ~3.0 mg cm^−2^. For half battery, sodium foil was used as the anode, and 1.0 M NaPF_6_ in PC containing 5 wt.% of FEC was employed as the electrolyte with glass fiber filter papers as the separator. The charge/discharge tests and Galvanostatic intermittent titration technique (GITT) were carried out on a Land CT2001A battery test system (Land, Wuhan, China) in a voltage range of 1.8–4.3 V at 25 °C. GITT was measured by applying the repeated current pulses for 30 min at a current density of 10 mA g^−1^, followed by relaxation for 1.5 h. CV experiments were performed within the same potential range at 0.1 mV s^−^^1^. CV was carried out on a Solartron 1470E electrochemical workstation in a voltage range of 1.8–4.3 V. The full battery was assembled with P2-Na_0.612_K_0.056_MnO_2_ as the cathode, HC as the anode, and 1.0 M NaPF_6_ in PC containing 5 wt.% of FEC as the electrolyte, respectively. In order to supplement sufficient Na^+^ in HC before assembling full battery, the HC electrode was obtained by disassembling the half battery in the glove box after half-discharging at 50 mA g^−1^. The mass ratio of Na_0.612_K_0.056_MnO_2_/HC is around 1.2, corresponding to 4% excess of anode capacity. The charge/discharge and CV tests were carried out at 25 °C in a voltage range of 1.0–4.3 V.

### Material characterizations

The chemical composition of the electrodes was determined by an inductively coupled plasma-optical emission spectrometer (ICP-OES, SPECTRO-BLUE). XRD was collected on Rigaku SmartLab equipped with a Cu Kα radiation source. Rietveld refinement was conducted with the Fullprof software^46^. The PDF analysis was carried out based on X-ray scattering data measured at the 11-ID-B beamline of the Advanced Photon Source at Argonne National Laboratory. High energy X-rays (*λ* = 0.1173 Å) were used to collect data to high values of momentum transfer (*Q* ~ 30 Å^−1^). Diffraction images were integrated within fit2D to obtain one-dimensional diffraction data^47^. Pair distribution functions, *G*(r), were extracted from the data within PDFgetX3^48^, after correcting for background and Compton scattering. Mn K-edge X-ray absorption fine structure (XAFS) results were collected at the beamline 8C in Pohang Accelerator Laboratory (PAL). The XAFS data were collected by fluorescence mode and processed through Demeter software package^49^. The extended x-ray absorption fine structure (EXAFS) region was *k*^3^ weighted and Fourier-transformed in *k*-ranges of 3.0–10.0 Å^−1^. Schematic representations of the synthesized samples were obtained by using the VESTA software^50^. The morphology was examined by SEM (JEOL JSM-7500F, AEMC, 5 kV). The HRTEM images, SAED patterns and EDS mappings were recorded by TEM (FEI Taosl F200X G2, AEMC, 200 kV). HAADF and ABF images were obtained on a JEM-AR200F STEM with an accelerating voltage of 200 kV with cold filed-emission gun and double hexapole Cs correctors (CEOS GmbH, Heidelberg, Germany). Dynamic secondary ion mass spectroscopy (SIMS) was carried out on a Cameca IMS-5FE7 system using Cs^+^ primary ions with a beam current of 3 nA. Solid-state ^39^K NMR spectra were obtained on a JNM-ECZ600R widebore spectrometer.

### DFT calculation

The crystal structures of Na_0.500_K_0.055_MnO_2_, Na_0.220_K_0.055_MnO_2_, and Na_0.275_MnO_2_ were simulated using Vienna ab-initio simulation package (VASP). The generalized gradient approximation (GGA) of exchange-correlation energy, Perdew-Burke-Eznerhof (PBE) functional, was used with its corresponding projector augmented-wave pseudopotentials^31–34^. A Hubbard *U* term of 3.9 is adopted to better describe the strong correlation of Mn 3*d* electrons. The slight difference between the simulated formulas and the obtained composition is due to that the number of K and Na atoms can only be integral in a unit cell, and such difference can be reasonably ignored. All optimization calculations adopted a 4 × 4 × 4 Monkhorst-Pack^51^ k-point grid and a cutoff energy of 400 eV, and a higher cutoff of 450 eV and a 6 × 6 × 4 k-point grid were applied for DOS and charge calculations for enhanced accuracy. First-principle molecular dynamics calculations, which adopt an NVT ensemble at 1000 K, were carried out using a single Γ point to balance the accuracy and cost. Atomic positions and cell vectors were fully optimized until all force components were less than 0.02 eV Å^−1^. The trajectory of MD simulation was further analyzed using Rigorous Investigation of Networks Generated using Simulations (R.I.N.G.S.) code to obtain MSD statistics. The COHP calculations were carried out using Local-Orbital Basis Suite towards Electronic-Structure Reconstruction (LOBSTER) code. The diffusion energy barriers are calculated using climbing-image nudged elastic band (CI-NEB) method with a single Γ point.

## Supplementary information

Supplementary Information

## Data Availability

The data that support the findings of this study are available from the corresponding author upon reasonable request.
