# Supplementary material for: Tuning local chemistry of P2 layered-oxide cathode for high energy and long cycles of sodium-ion battery
Source: Nat Commun. 2021 Apr 15;12:2256. doi: 10.1038/s41467-021-22523-3 (PMC8050090; doi:10.1038/s41467-021-22523-3)
Supplement: Supplementary file 1 — Supplementary Information [file 41467_2021_22523_MOESM1_ESM.pdf]

Supplementary Information

Tuning local chemistry of P2 layered-oxide cathode for high energy and long cycles of sodium-ion battery

Wang et al.

Supplementary Figures

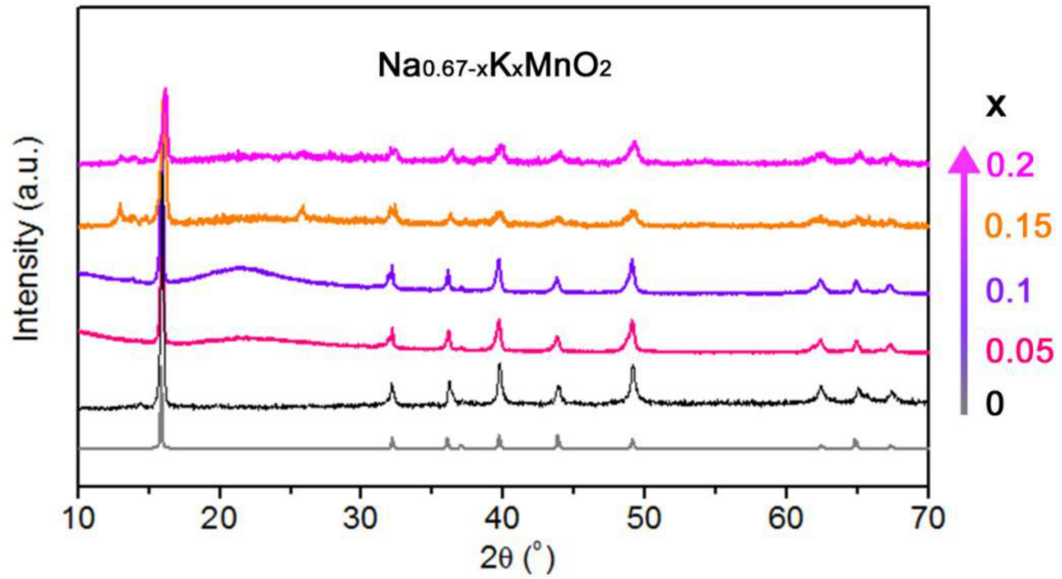

Supplementary Fig. 1 XRD patterns for the as-synthesized $Na_{0.67-x}K_xMnO_2$ ($0 \leq x \leq 0.2$) using sodium acetate precursor.

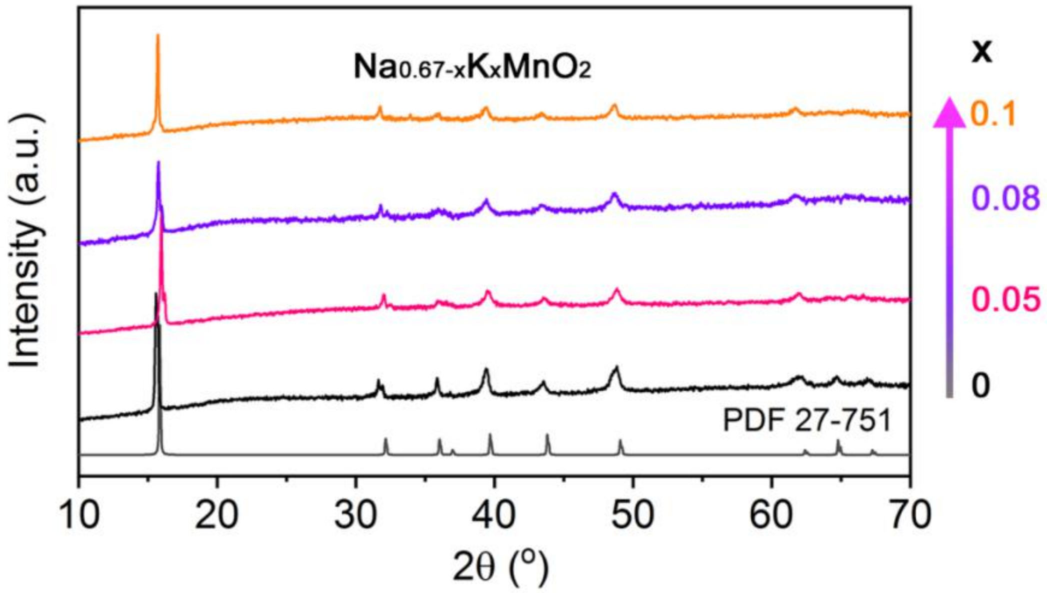

Supplementary Fig. 2 XRD patterns for the as-synthesized $Na_{0.67-x}K_xMnO_2$ ($0 \leq x \leq 0.1$) using sodium carbonate precursor.

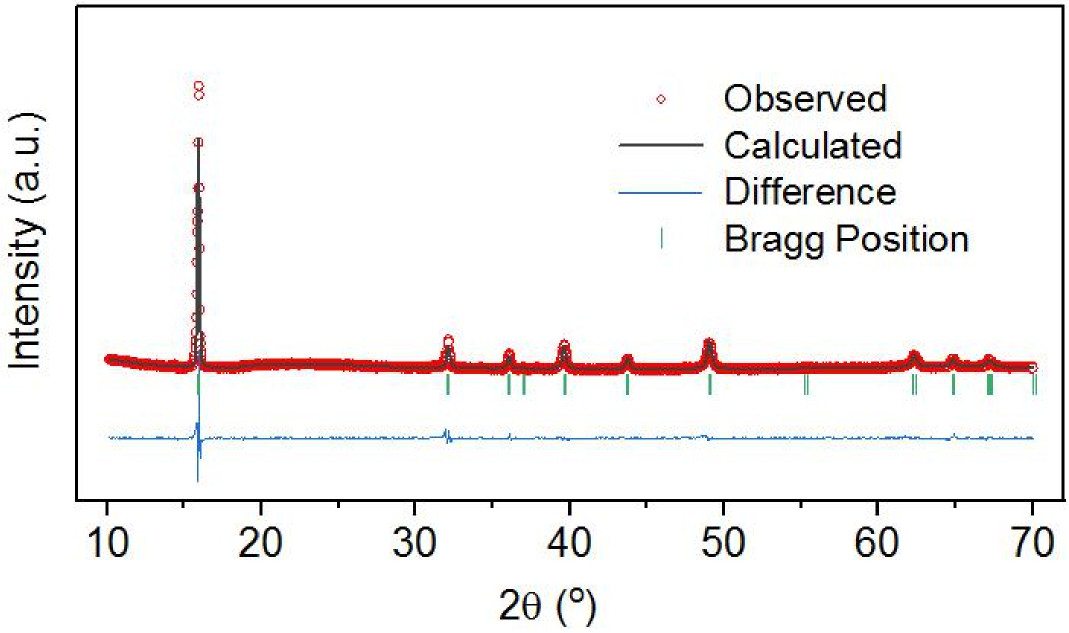

Supplementary Fig. 3 XRD Rietveld refinement pattern for the as-synthesized $Na_{0.706}MnO_2$.

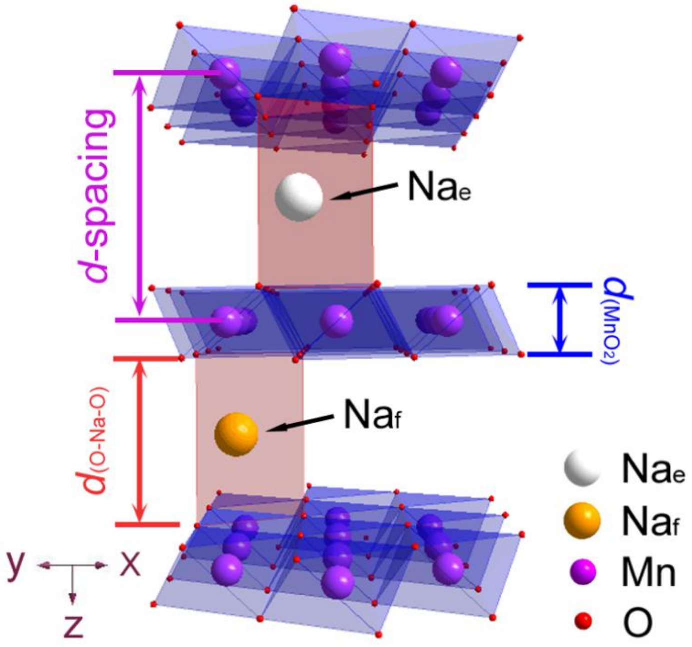

Supplementary Fig. 4 Structure of $Na_{0.706}MnO_2$ in the *$P6_3/mmc$* space group viewed along the y axis. It shows the typical Na_e (edge-sharing), Na_f (face-sharing) sites, *d*-spacing, $d_{(Na\text{-}O\text{-}Na)}$ and d_{MnO2}.

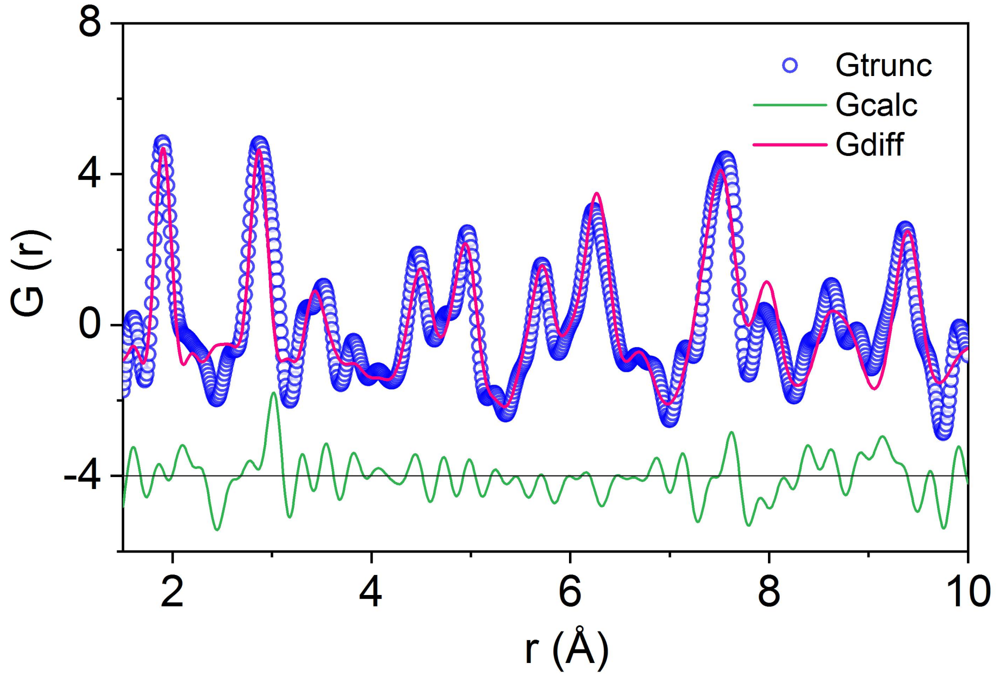

Supplementary Fig. 5 PDF pattern of $Na_{0.706}MnO_2$.

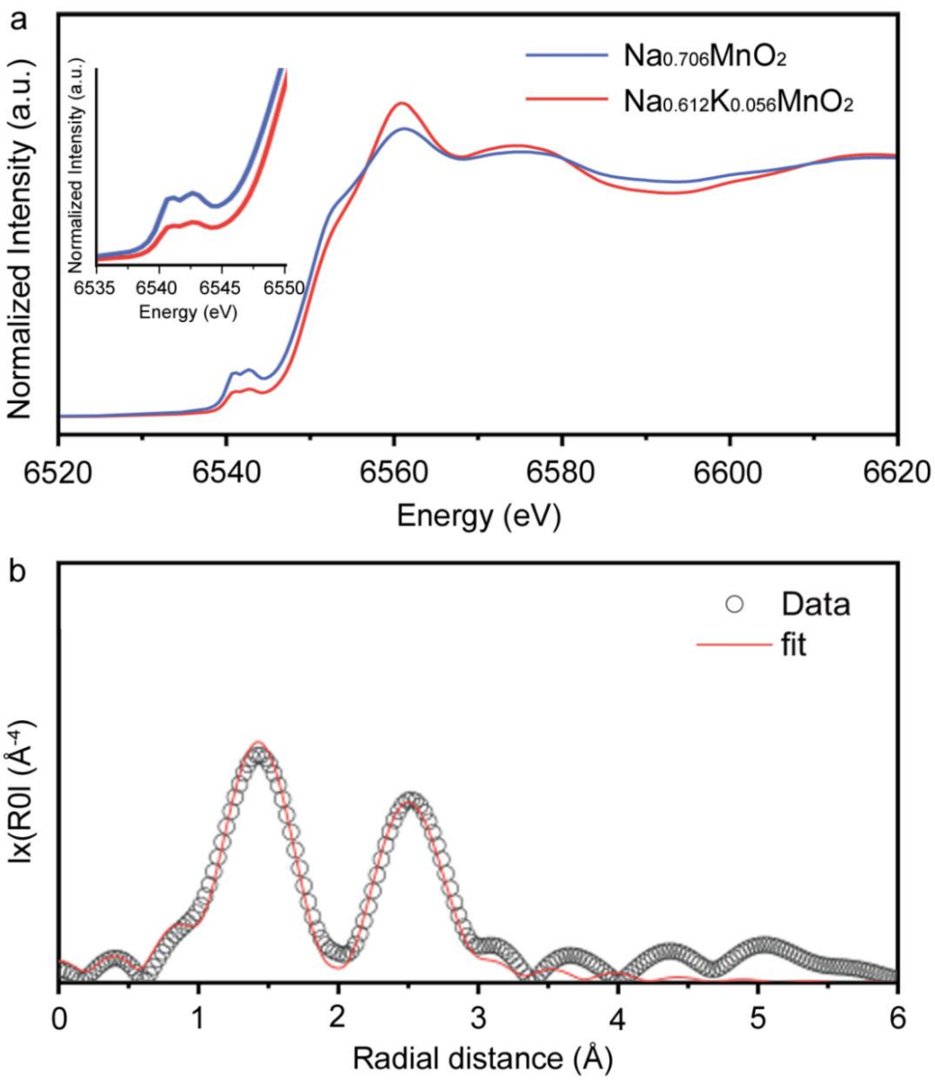

Supplementary Fig. 6 a, XANES spectra at the Mn K-edge for $Na_{0.612}K_{0.056}MnO_2$ and $Na_{0.706}MnO_2$. **b**, Fitting of Mn K-edge FT-EXAFS spectra of $Na_{0.706}MnO_2$. The magnified pre-edge region is shown in Supplementary figure 6a inset. In the pre-edge region, two peaks are observable, and these Mn pre-edge peaks look similar for both $Na_{0.612}K_{0.056}MnO_2$ and $Na_{0.706}MnO_2$, which implies that Mn has the approximately same average oxidation states.

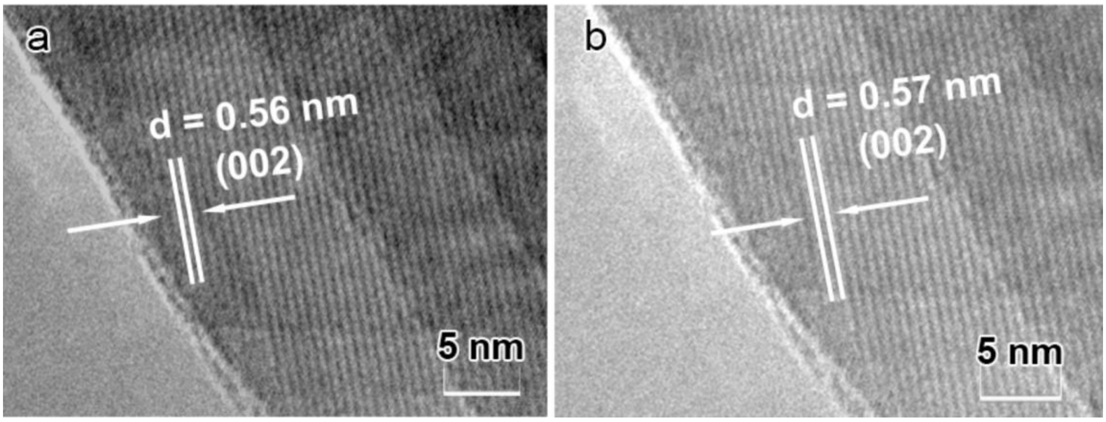

Supplementary Fig. 7 HRTEM images for the as-synthesized $Na_{0.612}K_{0.056}MnO_2$ (a) and $Na_{0.706}MnO_2$ (b).

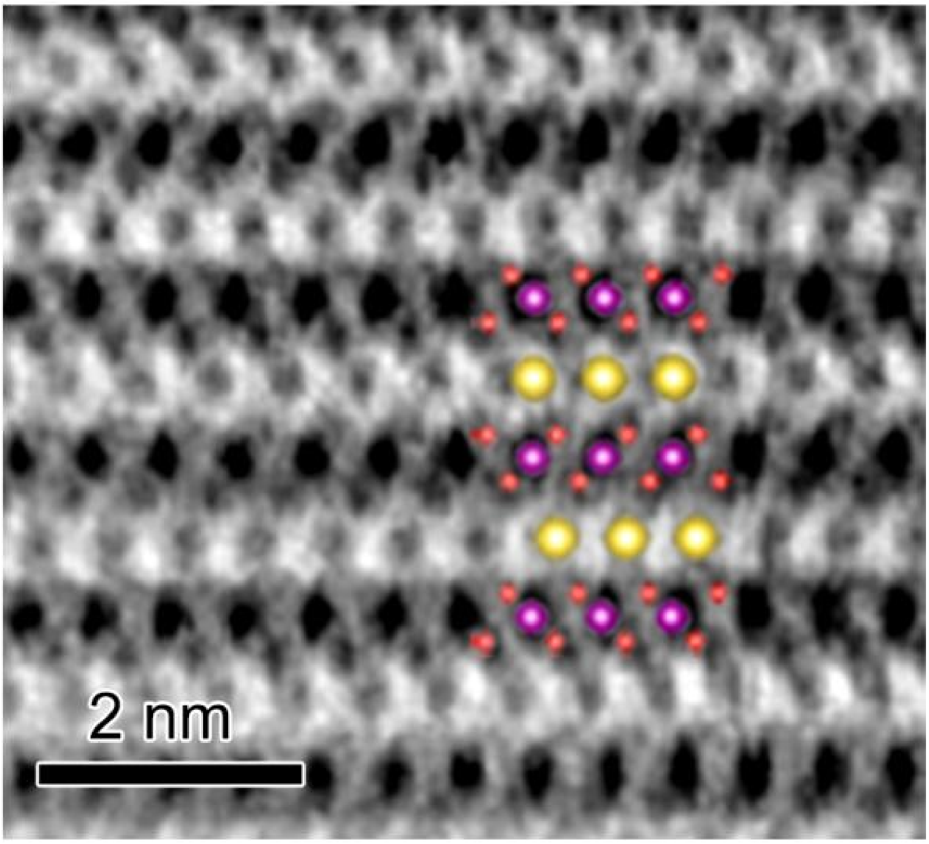

Supplementary Fig. 8 STEM-ABF image for the as-synthesized $Na_{0.602}K_{0.056}MnO_2$.

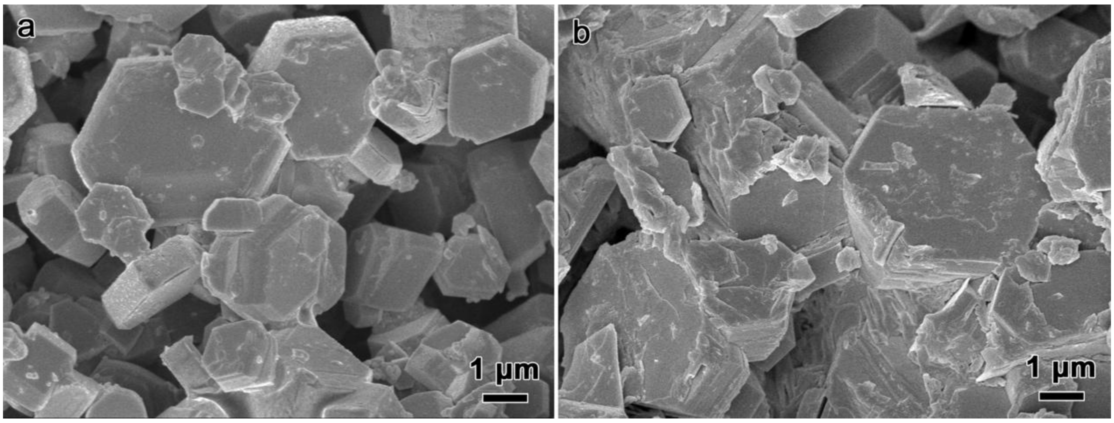

Supplementary Fig. 9 SEM images for the as-synthesized $Na_{0.612}K_{0.056}MnO_2$ (a) and $Na_{0.706}MnO_2$ (b).

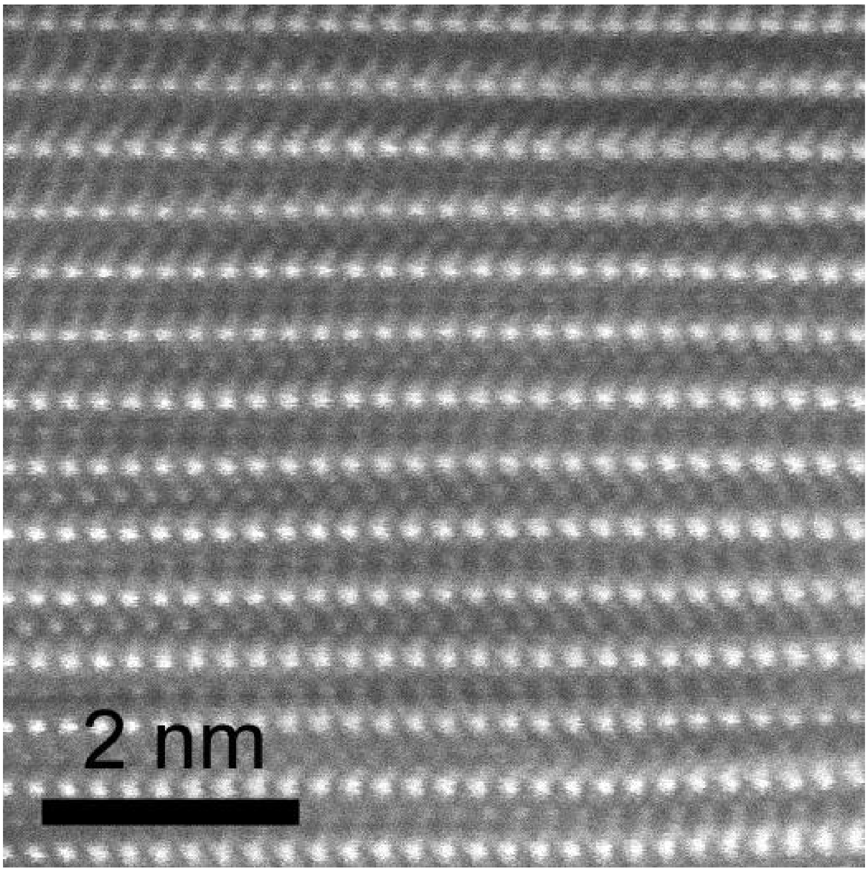

Supplementary Fig. 10 STEM-HAADF image for the as-synthesized $Na_{0.706}MnO_2$.

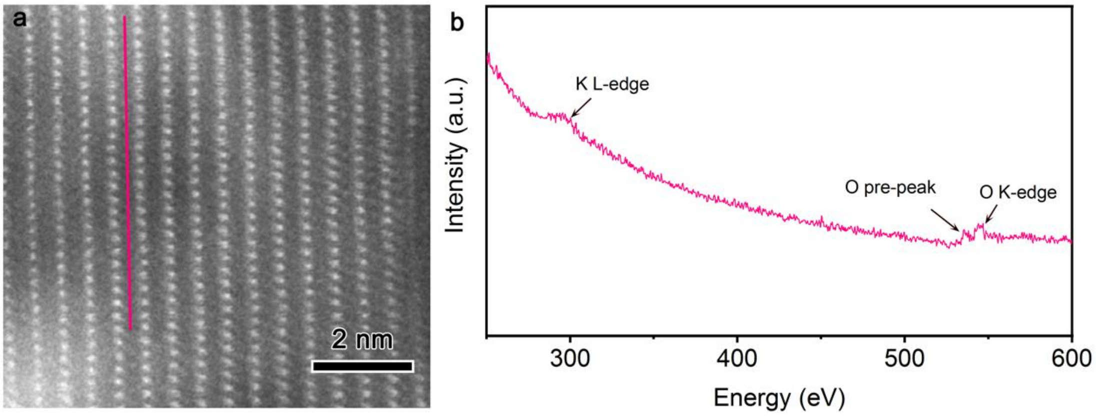

Supplementary Fig. 11 a, STEM-HAADF image of the as-synthesized $Na_{0.612}K_{0.056}MnO_2$ (b) and EELS profile scanned along the pink line in (a).

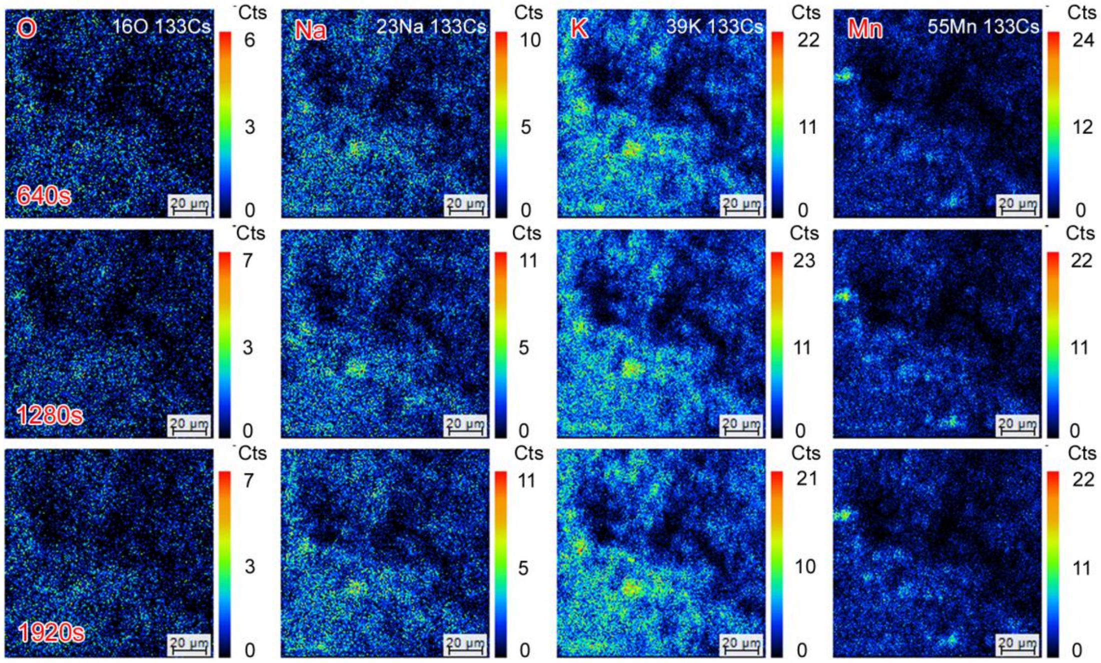

Supplementary Fig. 12 Elemental secondary ion mass spectrometry (SIMS) mapping of the pristine $Na_{0.612}K_{0.056}MnO_2$.

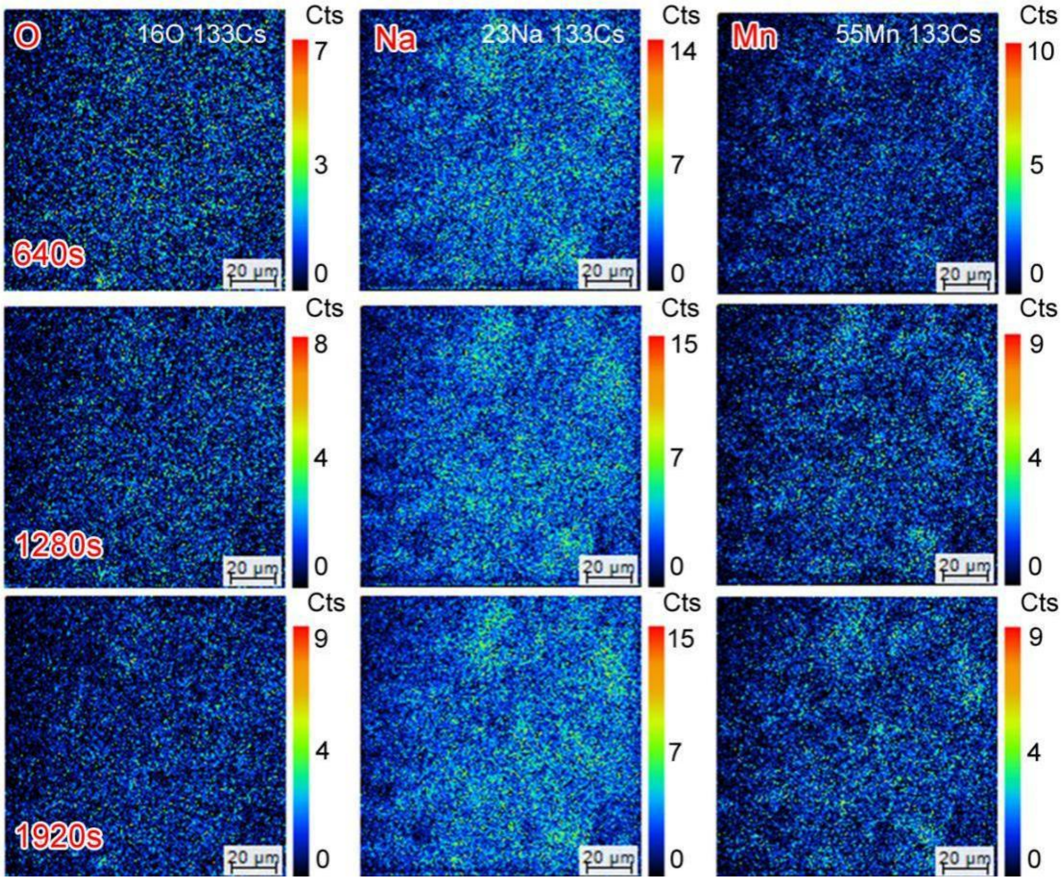

Supplementary Fig. 13 Elemental secondary ion mass spectrometry (SIMS) mapping of the pristine $Na_{0.706}MnO_2$.

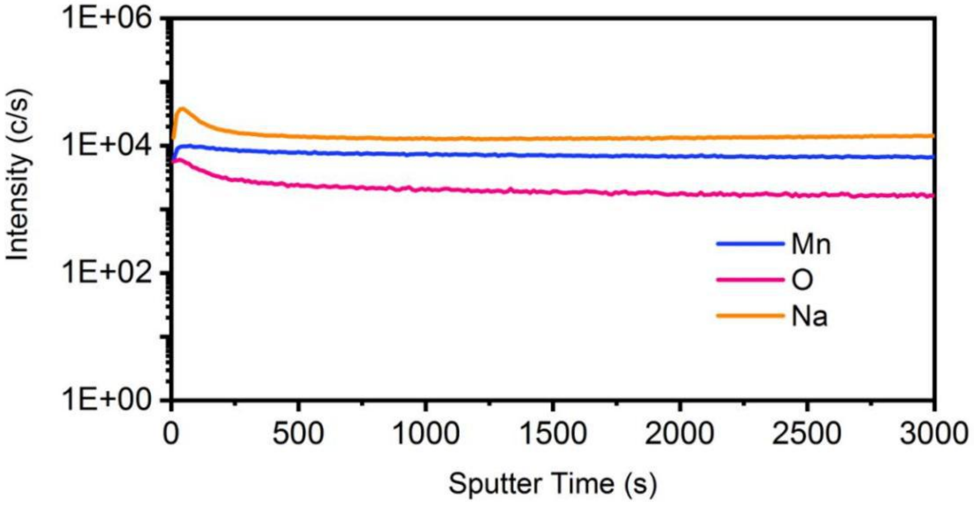

Supplementary Fig. 14 Sputter depth profiles of $Na_{0.706}MnO_2$.

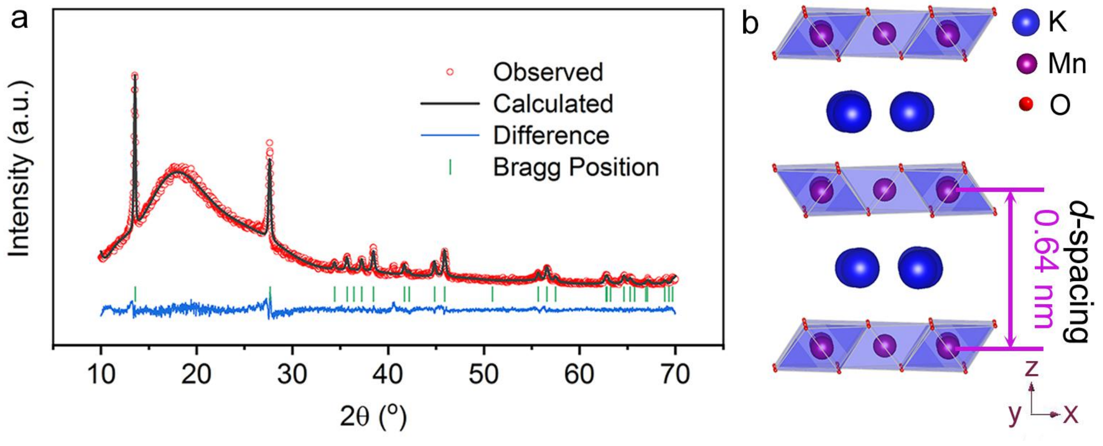

Supplementary Fig. 15 a, XRD Rietveld refinement pattern for $K_{0.67}MnO_2$. b, Structure of layered $K_{0.67}MnO_2$ in the *ccmm* space group.

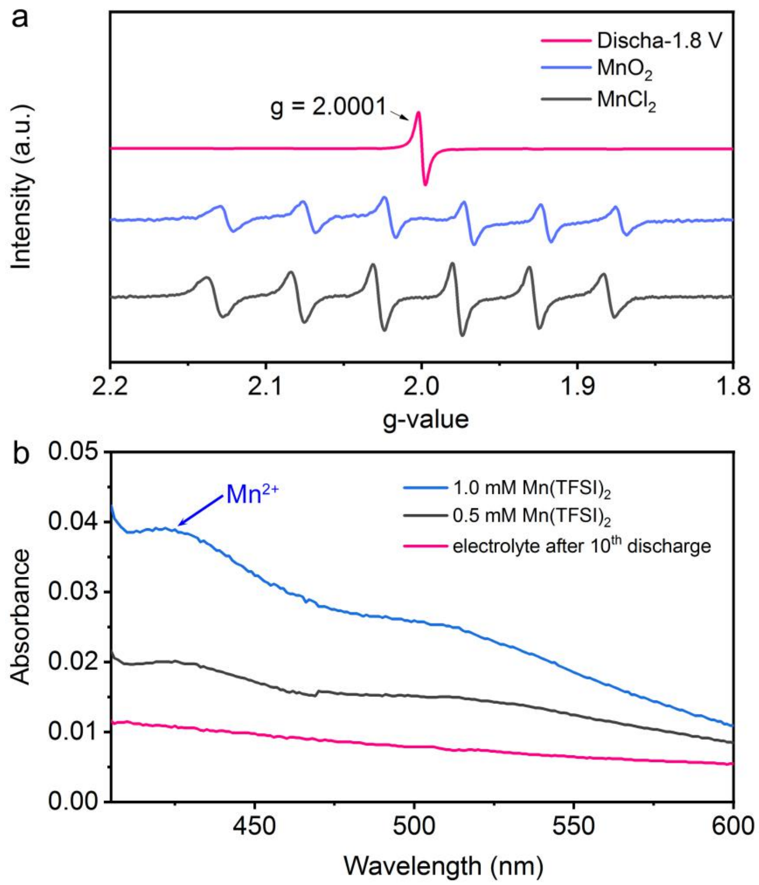

Supplementary Fig. 16 a, Electron paramagnetic resonance (EPR) spectra of discharged electrodes of P2-$Na_{1.015}K_{0.056}MnO_2$. b, Ultraviolet (UV) spectrum of electrolytes after 10th discharge of P2-$Na_{0.612}K_{0.056}MnO_2$ as well as 0.5 and 1.0 mM $Mn(TFSI)_2$. It was further confirmed that only Mn^{3+} exists in the full discharge state without Mn^{2+}. The EPR signal observed at *g*=2.0001 in $Na_{1.015}K_{0.056}MnO_2$ is attributed to the oxygen vacancies in the material surface.

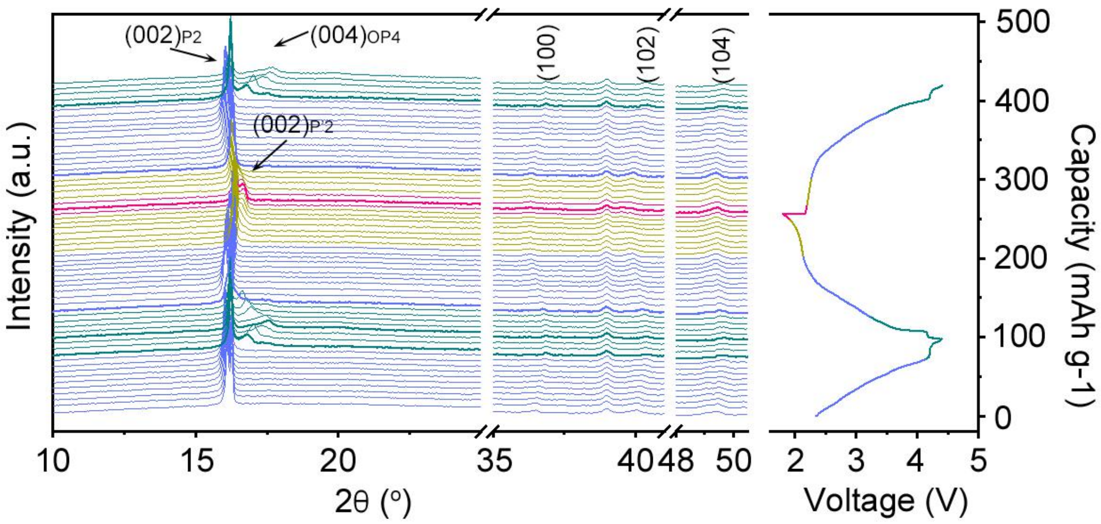

Supplementary Fig. 17 ***In situ*** **XRD patterns collected during the first charge/discharge and the second charge of the $Na_{0.706}MnO_2$ electrodes.** The (004) peak of OP4 phase appears at 17.5°, and the (002) peak of P'2 phase appears at 17.0°. It suggests that P2-$Na_{0.706}MnO_2$ involves the phase transition of P2→OP4 at high charge voltages during charge and P2→P'2 during discharge.

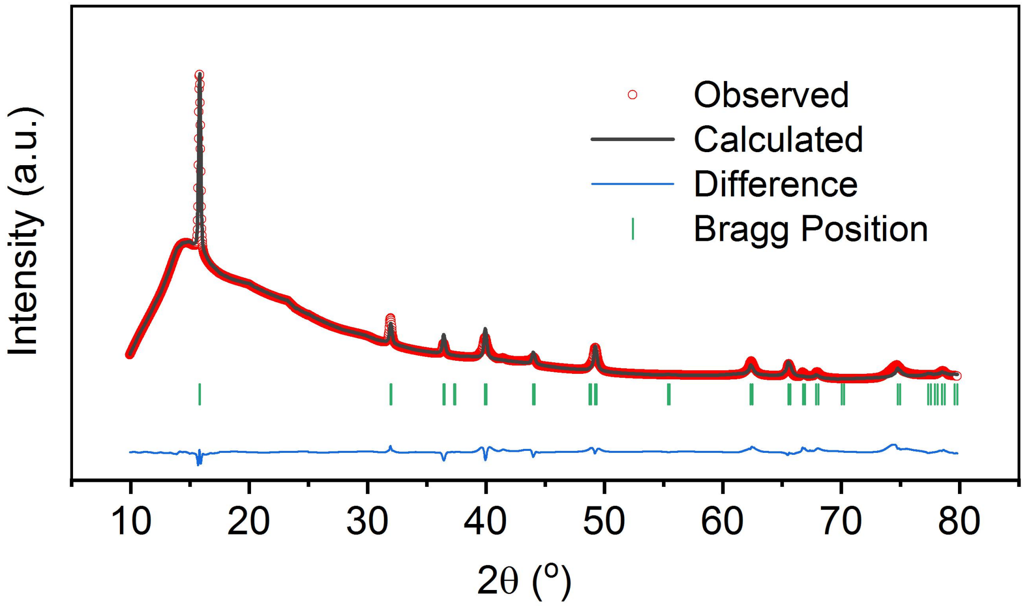

Supplementary Fig. 18 XRD Rietveld refinement pattern of $Na_{0.114}K_{0.056}MnO_2$.

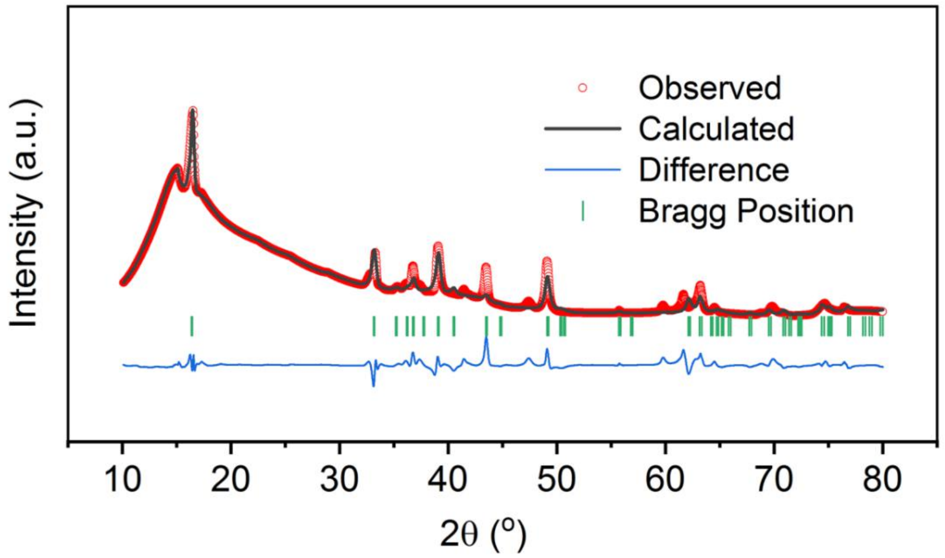

Supplementary Fig. 19 XRD Rietveld refinement pattern of $Na_{1.015}K_{0.056}MnO_2$.

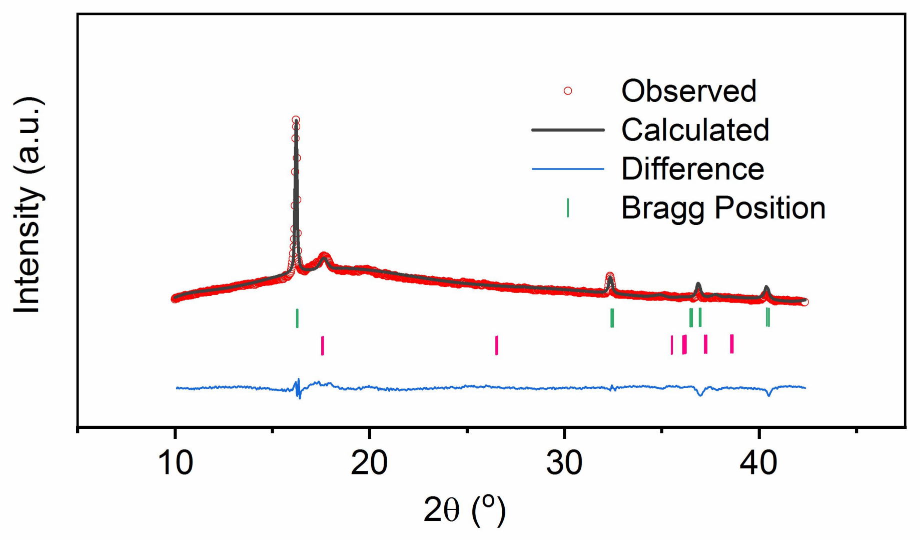

Supplementary Fig. 20 XRD Rietveld refinement pattern of $Na_{0.327}MnO_2$.

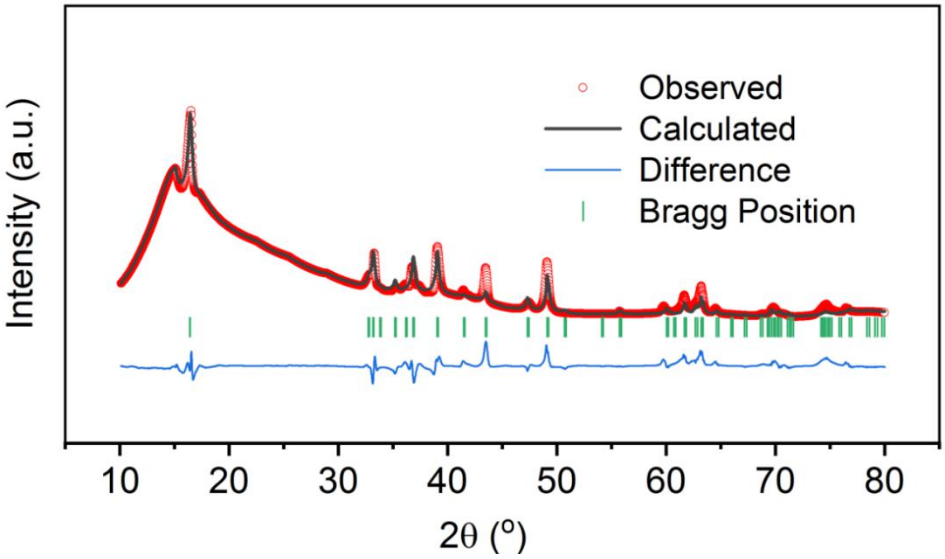

Supplementary Fig. 21 XRD Rietveld refinement pattern of $Na_{0.998}MnO_2$.

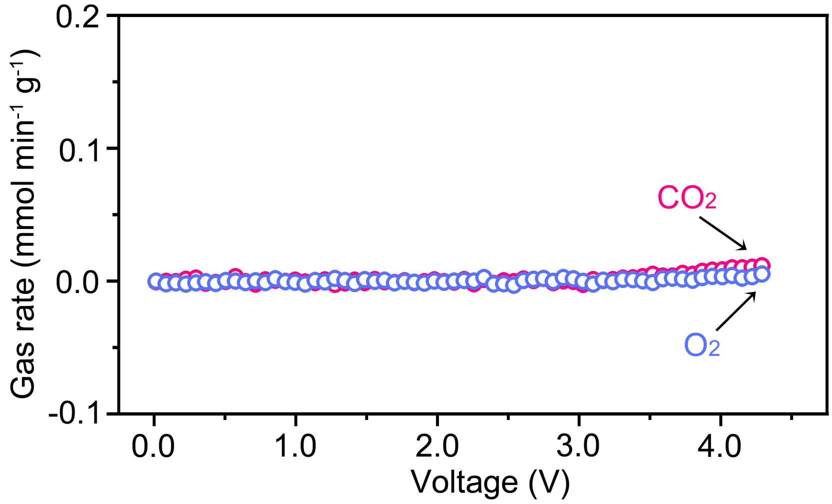

Supplementary Fig. 22 ***In situ*** **differential electrochemical mass spectrometry (DEMS) results of gas evolution rates of CO_2 and O_2 (unit: mmol min^{-1} g^{-1}).**

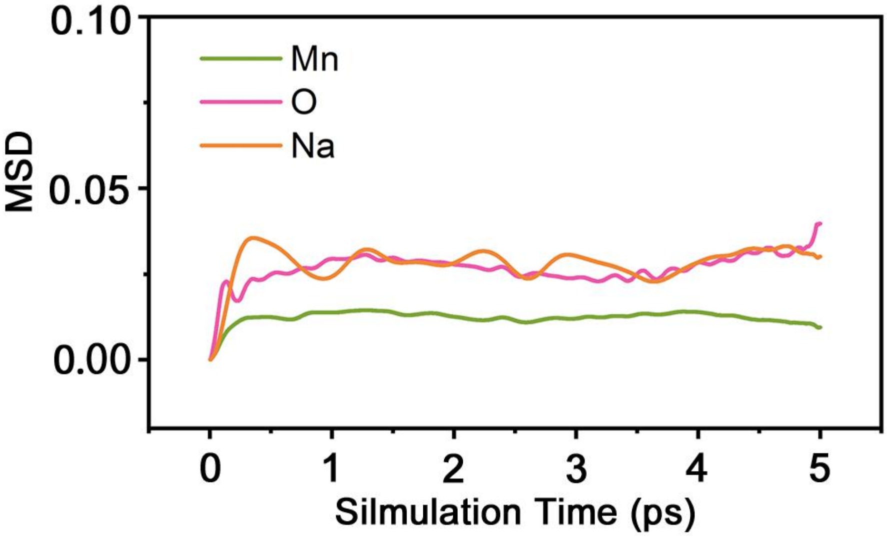

Supplementary Fig. 23 MSD of Mn, Na, and O as functions of time in $Na_{0.555}MnO_2$.

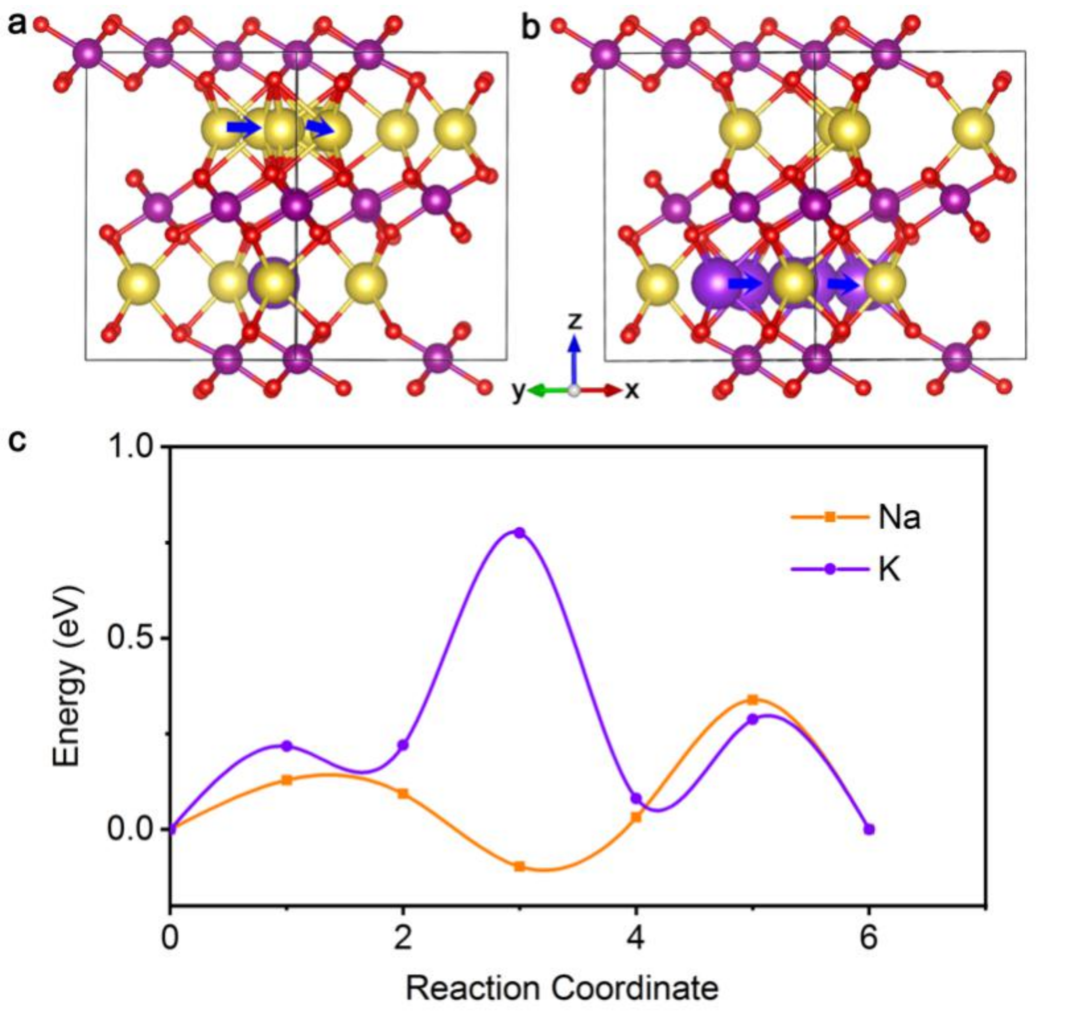

Supplementary Fig. 24 Diffusion path for Na^+ (a) and K^+ (b) and their migration energy barrier (c) in $Na_{0.612}K_{0.056}MnO_2$.

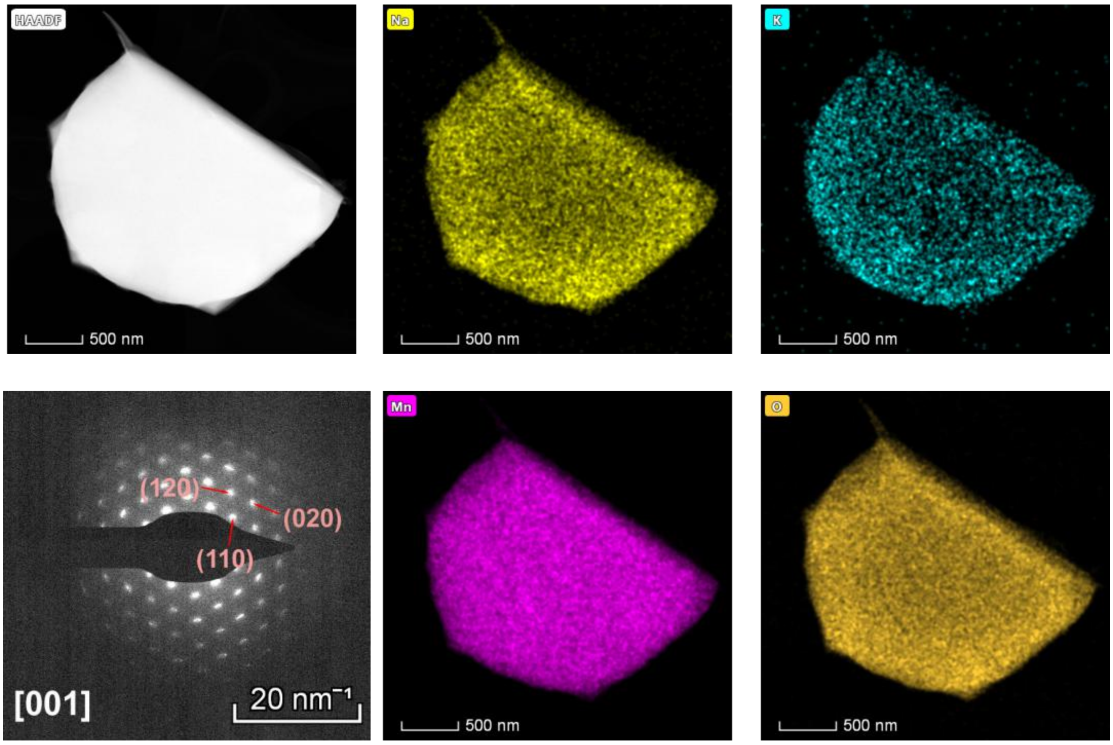

Supplementary Fig. 25 TEM elemental mappings and selected area electron diffraction (SAED) of the as-synthesized $Na_{0.612}K_{0.056}MnO_2$. SAED implies a pure P2 phase and the elemental mappings show uniform distribution of Na, K, Mn, and O in pristine $Na_{0.612}K_{0.056}MnO_2$.

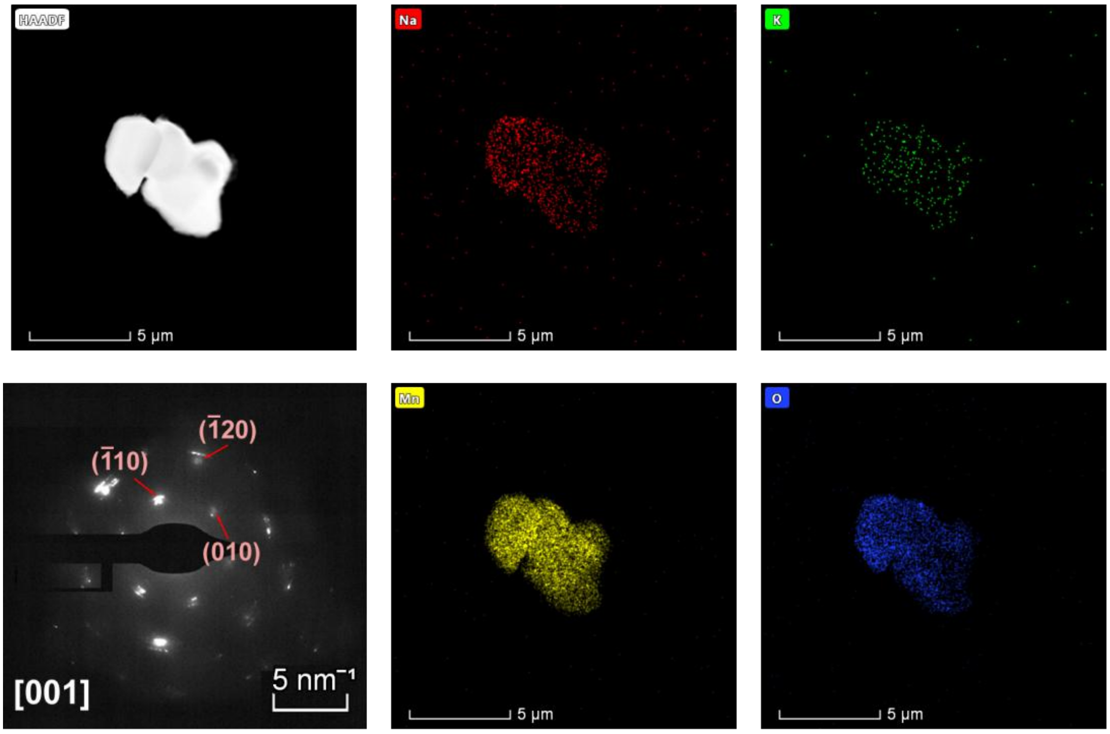

Supplementary Fig. 26 TEM elemental mappings and SAED of $Na_{0.612}K_{0.056}MnO_2$ after the first charge. SAED implies that a pure P2 phase was maintained after charging. The elemental mappings show uniform distribution of Na, K, Mn, and O after charge.

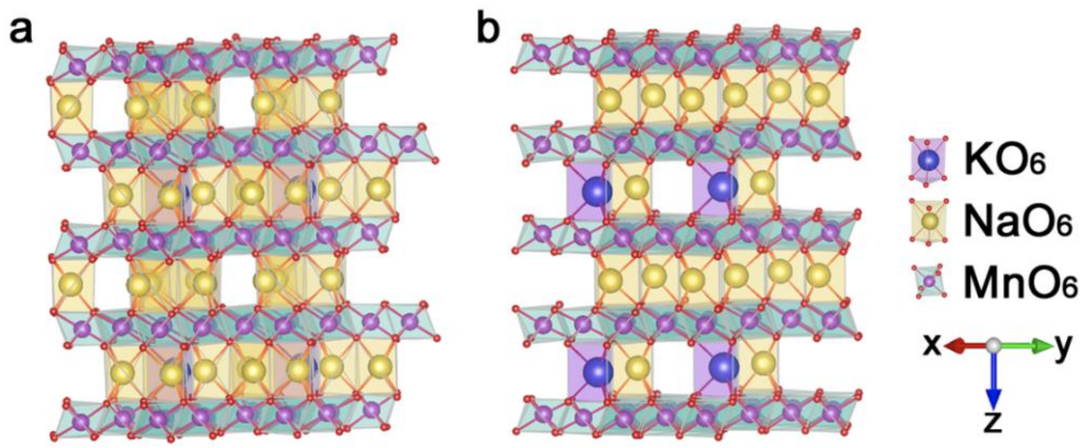

Supplementary Fig. 27 Optimized structure of P2-$Na_{0.500}K_{0.055}MnO_2$ (a) and P2-$Na_{0.220}K_{0.055}MnO_2$ (b).

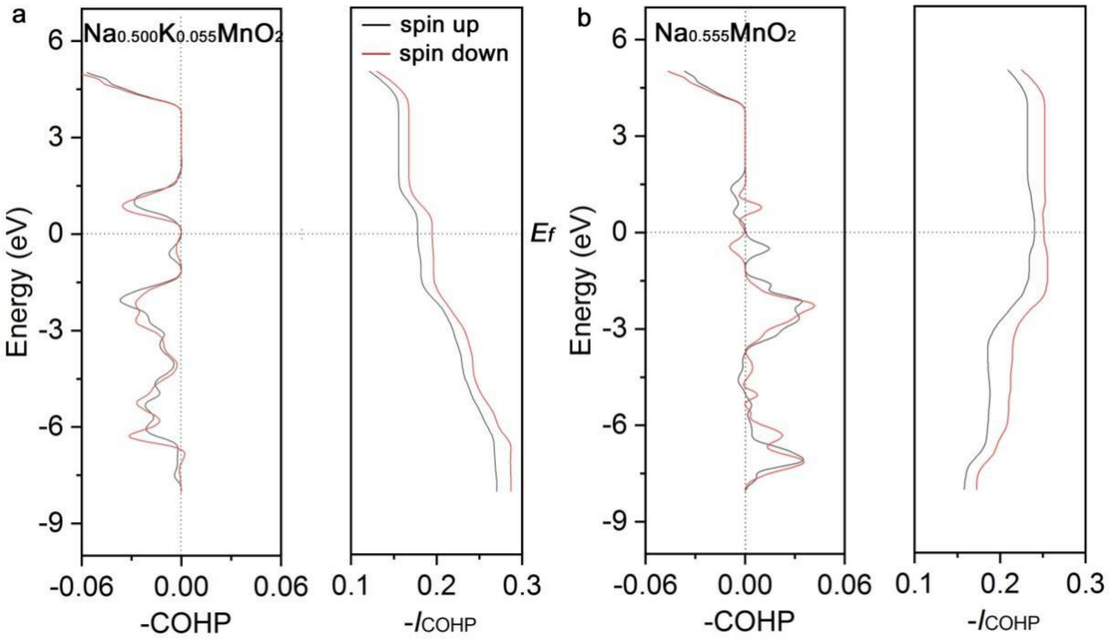

Supplementary Fig. 28 -COHP and corresponding integrated (-I_{COHP}) curves for K-O in $Na_{0.500}K_{0.055}MnO_2$ (a) and Na-O in $Na_{0.555}MnO_2$ (b).

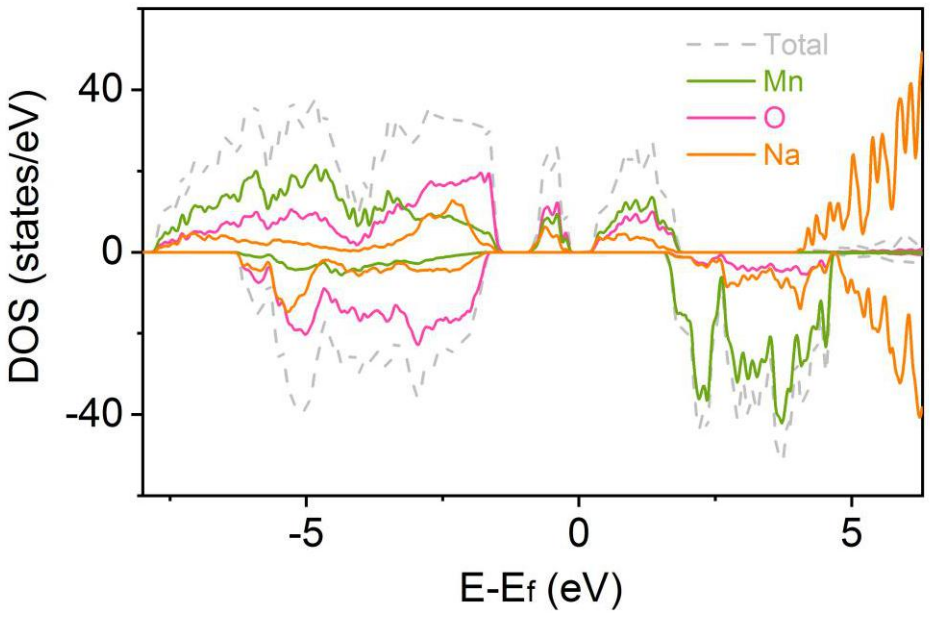

Supplementary Fig. 29 tDOS of $Na_{0.555}MnO_2$ and pDOS of Na 3*s*, K 4*s*, O 2*p* and Mn 3*d* orbitals. The Fermi energy is set to 0 eV.

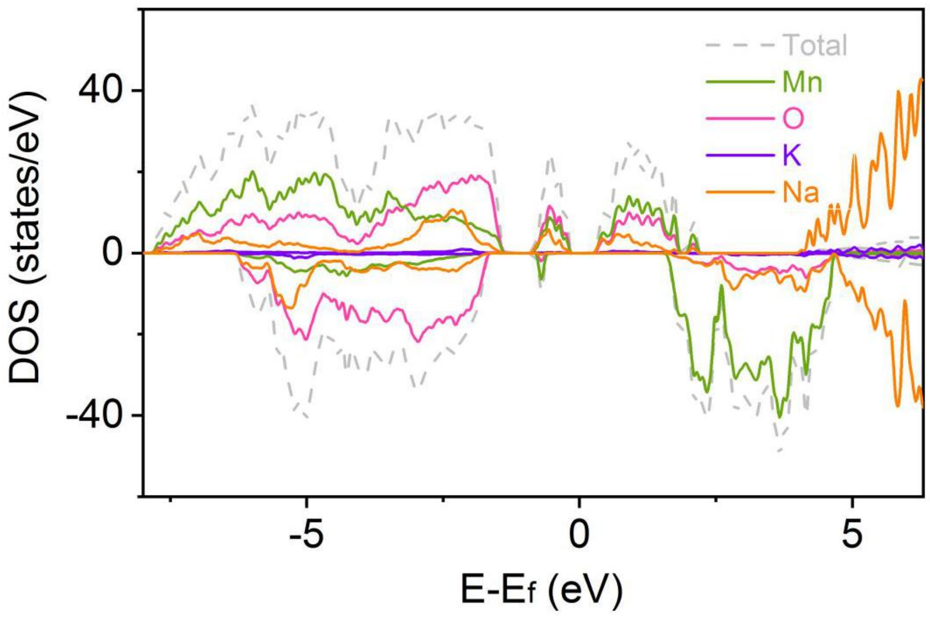

Supplementary Fig. 30 tDOS of $Na_{0.500}K_{0.055}MnO_2$ and pDOS of Na 3*s*, K 4*s*, O 2*p* and Mn 3*d* orbitals. The Fermi energy is set to 0 eV.

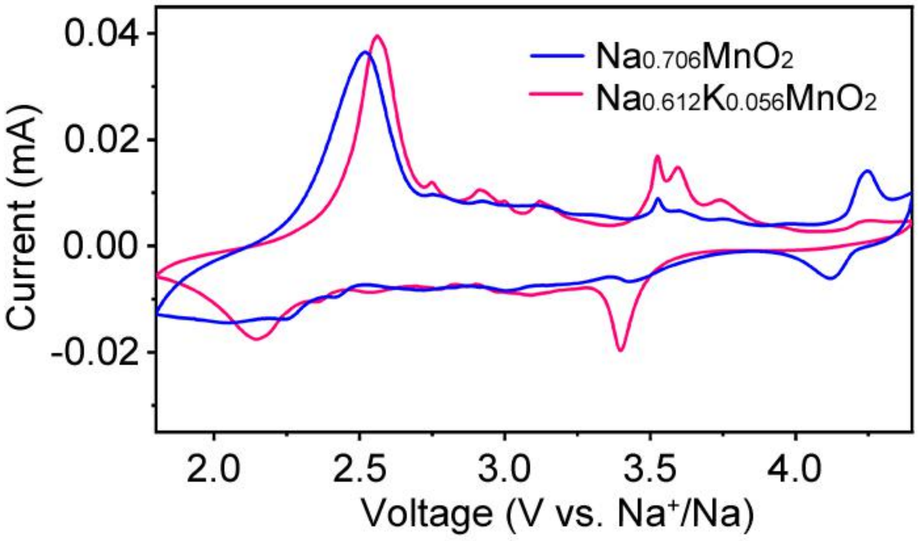

Supplementary Fig. 31 CV curves of $Na_{0.612}K_{0.056}MnO_2$ and $Na_{0.706}MnO_2$ at a scan rate of 0.1 mV s^{-1}.

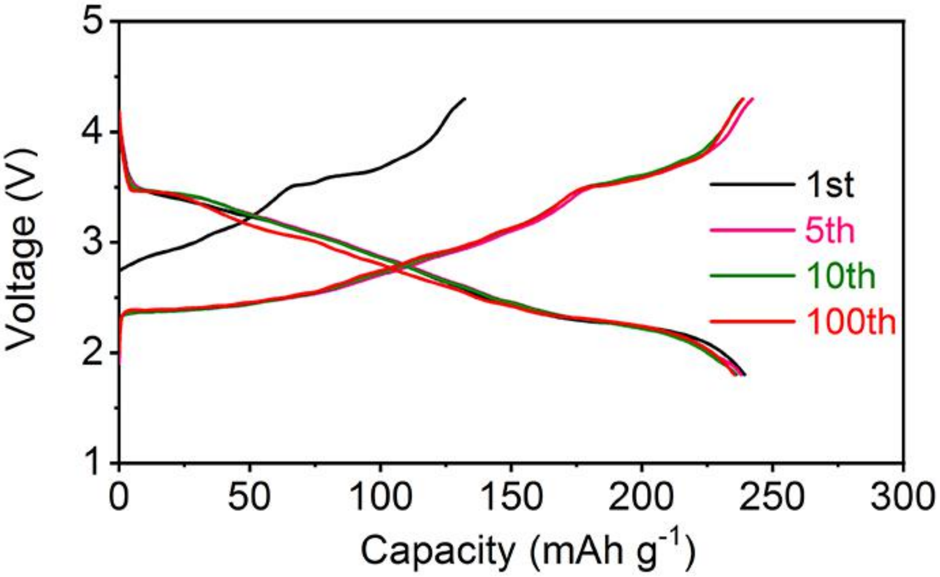

Supplementary Fig. 32 Selected charge/discharge curves of $Na_{0.612}K_{0.056}MnO_2$ at 50 mA g^{-1} during 100 cycles.

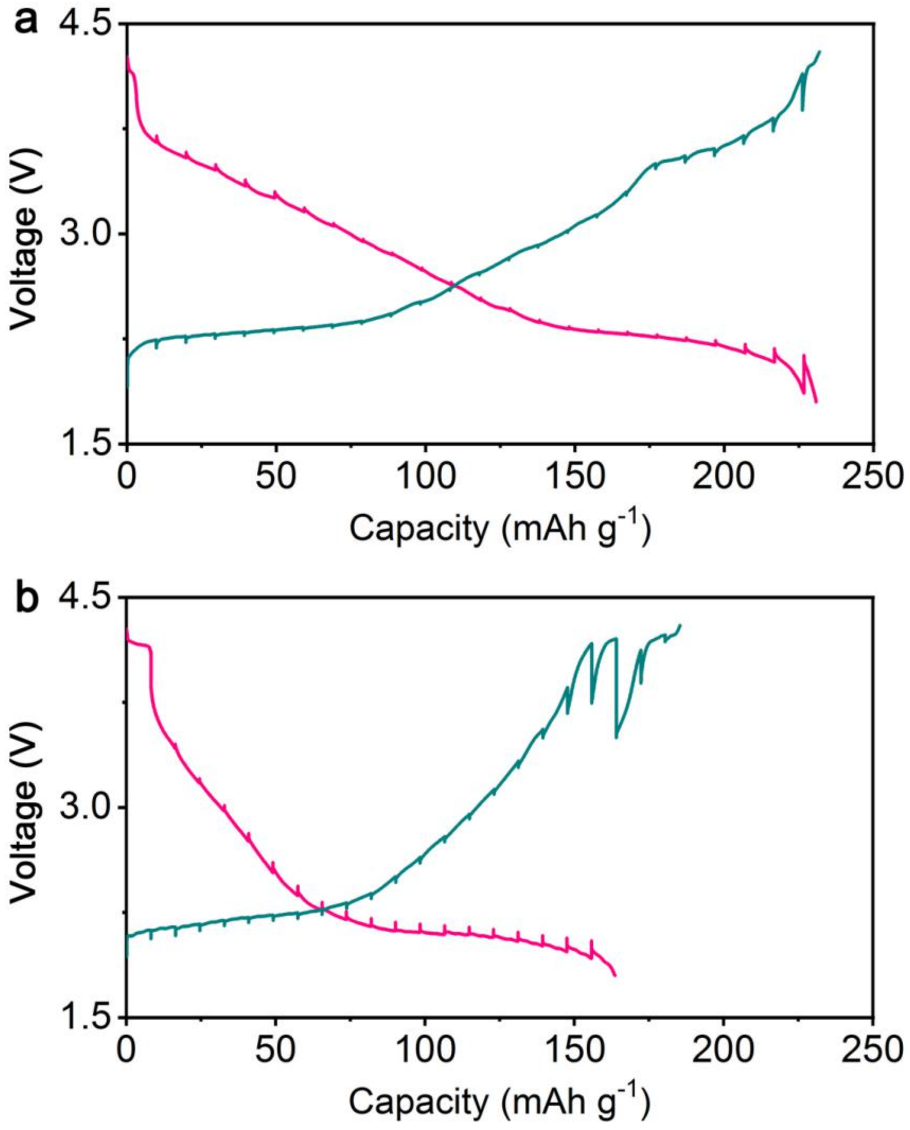

Supplementary Fig. 33 GITT curves of $Na_{0.612}K_{0.056}MnO_2$ (a) and $Na_{0.706}MnO_2$ (b) during the second cycle in the voltage range of 1.8-4.3 V.

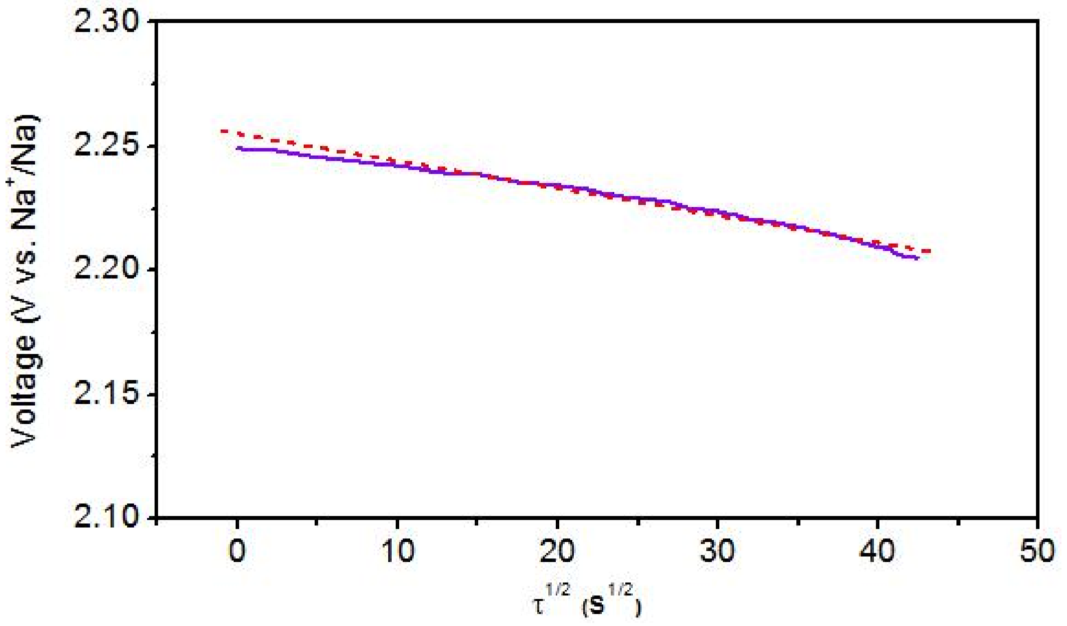

Supplementary Fig. 34 Linear fitting of $Na_{0.612}K_{0.056}MnO_2$ electrode at 2.25 V in the second discharge process.

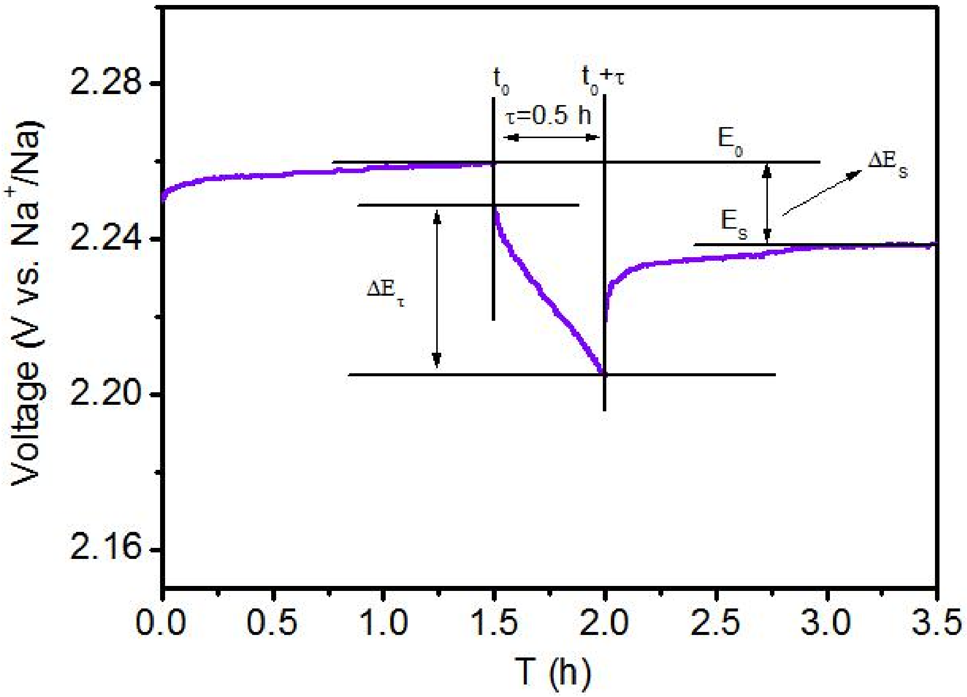

Supplementary Fig. 35 Current step diagrams of $Na_{0.612}K_{0.056}MnO_2$ electrode at 2.25 V in the second discharge process. The diffusion coefficient of Na^+ can be determined by applying the Fick's second law of diffusion, and the Equation is as following:

$$D_{Na^+} = \frac{4}{\pi\tau}\left(\frac{m_B V_m}{M_B A}\right)^2\left(\frac{\Delta E_S}{\Delta E_\tau}\right)^2$$

Where M_B, V_M, and m_B are molecular weight, molar volume, and mass of electrode material, respectively, A is geometric area of electrode.. $\triangle E_s$ and $\triangle E_\tau$ represent the change of quasi-equilibrium potential and battery voltage, respectively.

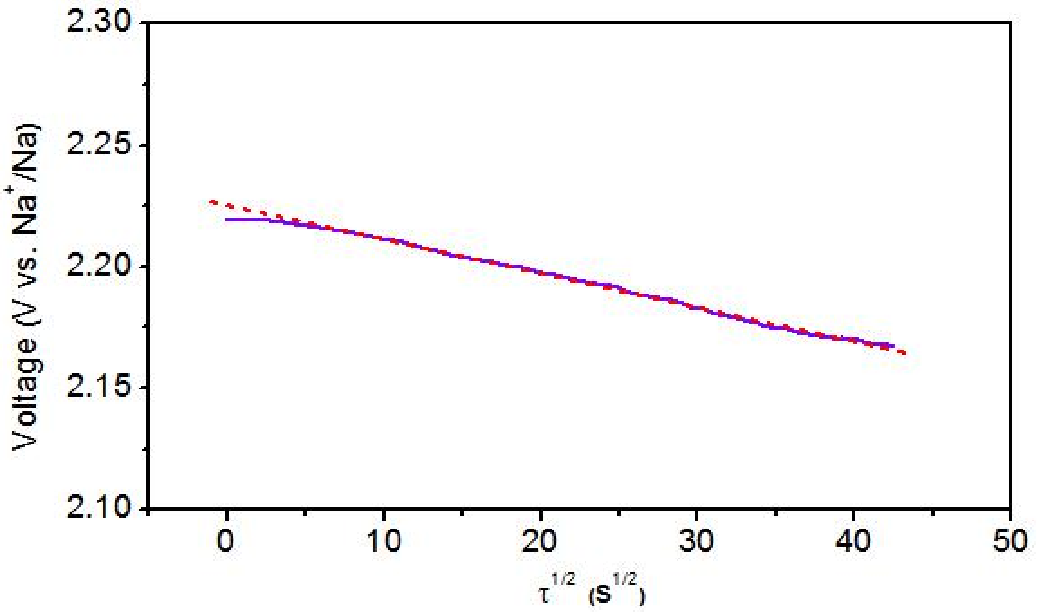

Supplementary Fig. 36 Linear fitting of $Na_{0.706}MnO_2$ electrode at 2.22 V in the second discharge process.

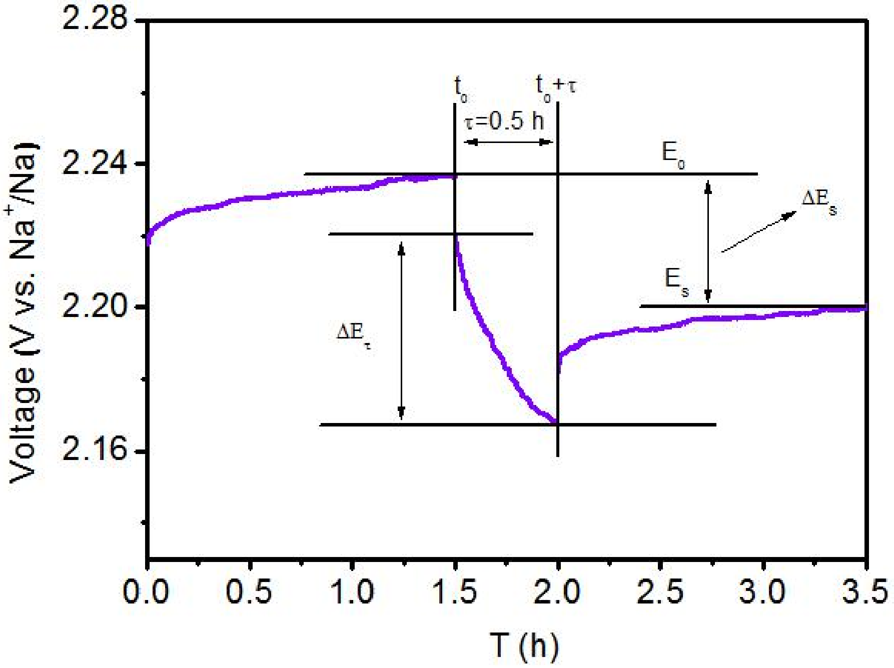

Supplementary Fig. 37 Current step diagrams of $Na_{0.706}MnO_2$ electrode at 2.22 V in the second discharge process.

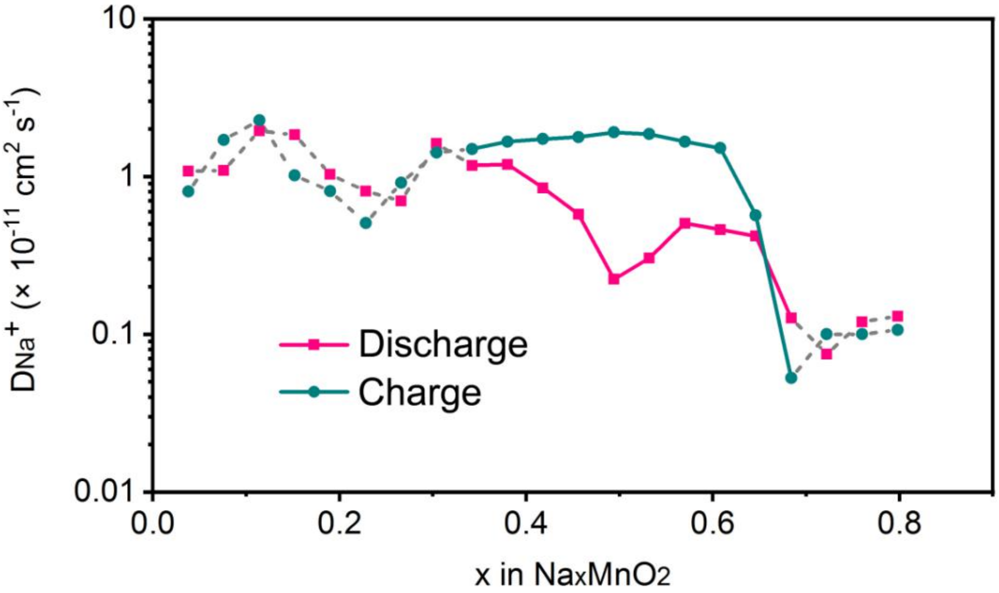

Supplementary Fig. 38 Variation of D_{Na+} as functions of x in Na_xMnO_2 determined by GITT, where the gray area represents the two-phase region.

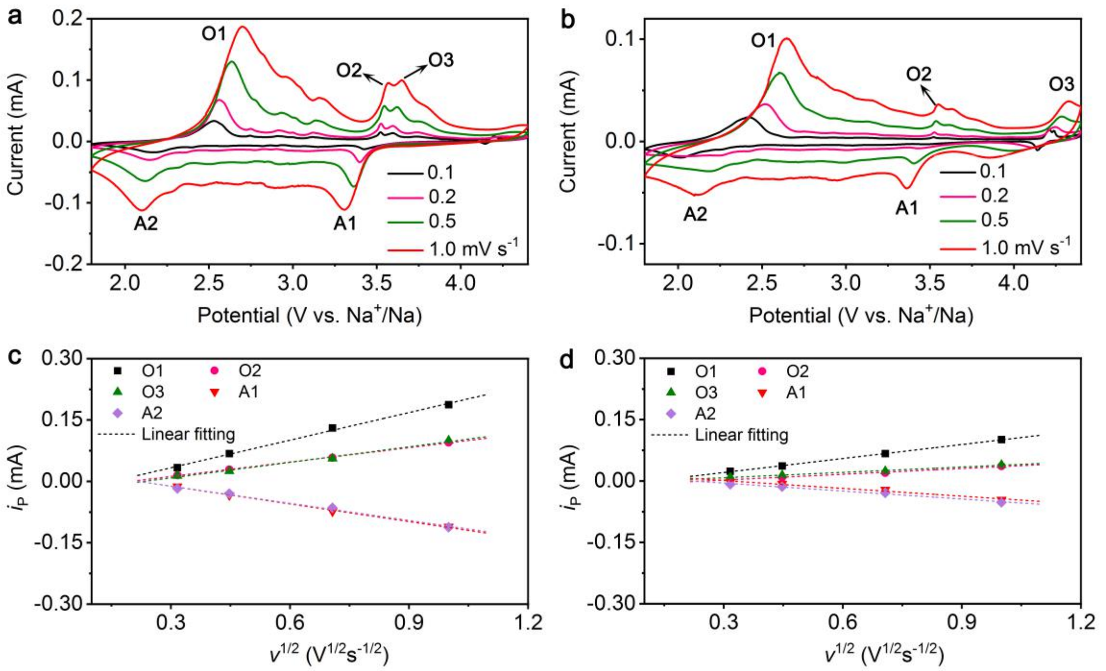

Supplementary Fig. 39 CV curves of $Na_{0.612}K_{0.056}MnO_2$ (**a**) and $Na_{0.706}MnO_2$ (**b**) at different scan rates of 0.1, 0.2, 0.5 and 1.0 mV s^{-1}. Liner relationship of the peak current (i_P) and the square root of scan rate ($v^{1/2}$) for peaks of O1, O2, O3, A1 and A2 in **Supplementary Fig. 39a** (**c**) and **Supplementary Fig. 39b** (**d**).

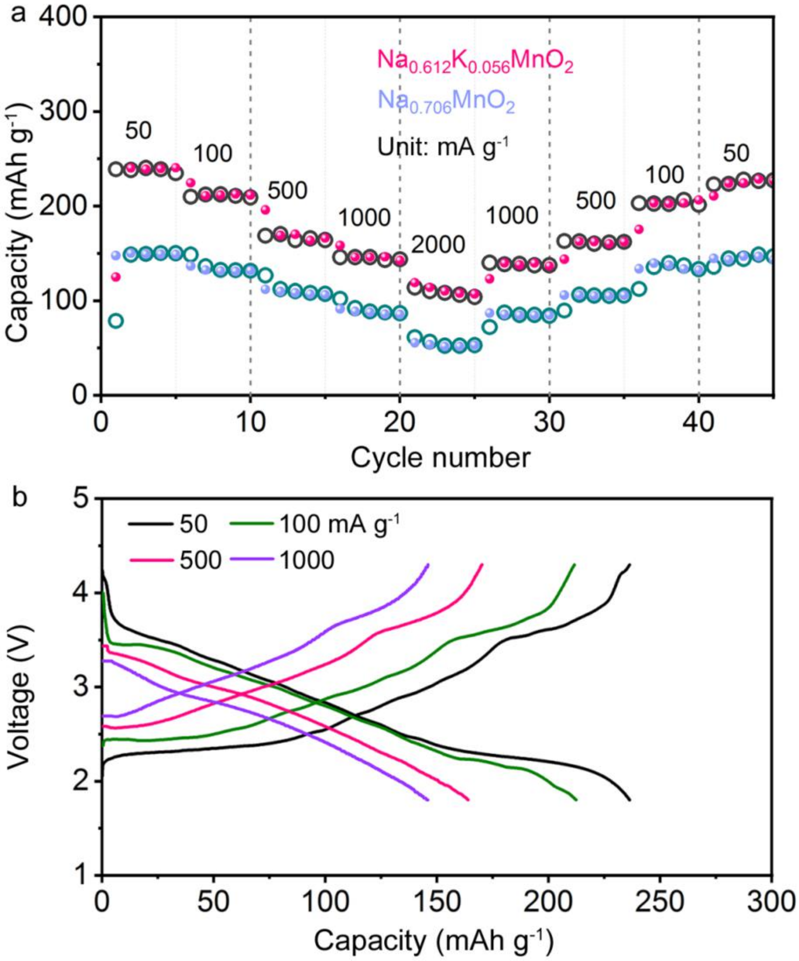

Supplementary Fig. 40 a, Rate performance of $Na_{0.612}K_{0.056}MnO_2$ and $Na_{0.706}MnO_2$. **b**, Typical discharge/charge curves of $Na_{0.612}K_{0.056}MnO_2$ at different current densities.

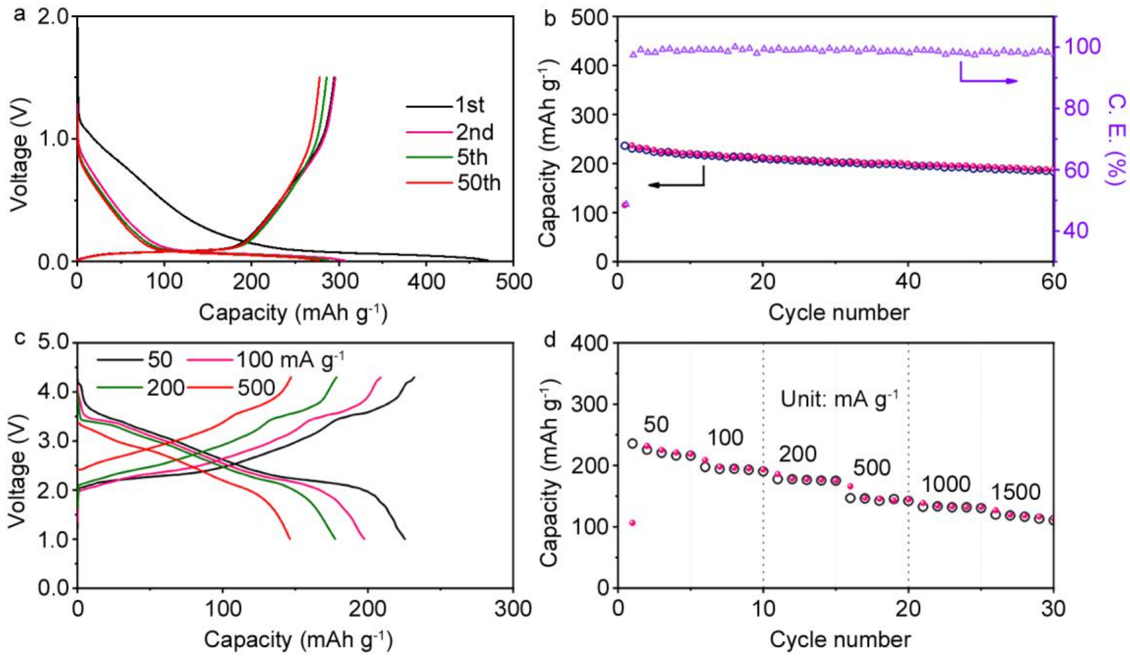

Supplementary Fig. 41 Electrochemical performance of HC electrode and HC//$Na_{0.612}K_{0.056}MnO_2$ full battery. a, Selected charge/discharge curves of HC at 50 mA g^{-1} during 50 cycles. **b**, Cycle performance of HC//$Na_{0.612}K_{0.056}MnO_2$ full battery at 50 mA g^{-1} in the voltage range of 1.0 V - 4.3 V. **c**, Typical discharge/charge curves of HC//$Na_{0.612}K_{0.056}MnO_2$ full battery at different current densities. **d**, Rate performance of HC//$Na_{0.612}K_{0.056}MnO_2$ full battery.

Supplementary Tables

Supplementary Table 1. ICP-OES of the synthesized P2-$Na_{0.612}K_{0.056}MnO_2$ and charged and discharged electrodes.

Elements	Atomic ratio		
	Pristine	Charged	Discharged
Na	0.612	0.116	1.010
K	0.056	0.055	0.055
Mn	1.00	1.00	1.00

Supplementary Table 2. Results of iodometric titration on discharged K-doped cathode.

	1	2	3
V ($Na_2S_2O_3$, mL)	12.60	12.65	12.65
Valence of Mn	3.298	3.303	3.303
δ	0.017	0.015	0.015

Supplementary Table 3. Structure parameters of $Na_{0.612}K_{0.056}MnO_2$ determined from the XRD Rietveld refinement.

Atom	Site	*g*	*x*	*y*	*z*
Na1	2*c*	0.320(1)	1/3	2/3	1/4
Na2	2*b*	0.294(2)	0	0	1/4
K1	2*b*	0.056(2)	1/3	2/3	1/4
Mn	2*a*	1.0	0.0	0.0	1/2
O1	4*f*	1.0	2/3	1/3	0.085(1)

Space group: $P6_3/m\ m\ c$ (194), $a = b = 2.868(3)$, $c = 11.134(10)$ Å, $\alpha = \beta = 90°$, $\gamma = 120°$, Vol = 79.310 Å^3 ($Z = 2$), $R_p = 2.93\%$, $R_{wp} = 3.96\%$, $\chi^2 = 2.987$. *g*: occupancy. *x*, *y*, and *z*: atomic coordinate.

Supplementary Table 4. Structure parameters of $Na_{0.706}MnO_2$ determined from the XRD Rietveld refinement.

Atom	Site	*g*	*x*	*y*	*z*
Na1	2*c*	0.323(11)	1/3	2/3	1/4
Na2	2*b*	0.388(11)	0	0	1/4
Mn	2*a*	1.0	0.0	0.0	1/2
O1	4*f*	1.0	2/3	1/3	0.088(1)

Space group: $P6_3/m\ m\ c$ (194), $a = b = 2.870(2)$, $c = 11.158(9)$ Å, $\alpha = \beta = 90^o$, $\gamma = 120^o$, Vol = 79.321 Å^3 ($Z = 2$), $R_p = 4.64\%$, $R_{wp} = 6.56\%$, $\chi^2 = 6.104$. *g*: occupancy. *x*, *y*, and *z*: atomic coordinate.

Supplementary Table 5. Synchrotron pair distribution function (PDF) refined structural parameters of $Na_{0.612}K_{0.056}MnO_2$.

Atom	Site	*g*	*x*	*y*	*z*
Na1	2*d*	0.298	1/3	2/3	1/4
Na2	2*b*	0.314	0	0	1/4
K1	2*d*	0.056	1/3	2/3	1/4
Mn	2*a*	1.0	0.0	0.0	1/2
O1	4*f*	1.0	2/3	1/3	0.0849(6)

Space group: $P6_3/m\ m\ c$ (194), $a = b = 2.8696$ Å, $c = 11.1243$ Å, $\alpha = \beta = 90°$, $\gamma = 120°$, Vol = 79.331 Å^3, $\chi^2 = 2.3524$. *g*: occupancy. *x*, *y*, and *z*: atomic coordinate.

Supplementary Table 6. Synchrotron pair distribution function (PDF) refined structural parameters of $Na_{0.706}MnO_2$.

Atom	Site	*g*	*x*	*y*	*z*
Na1	2*d*	0.350	1/3	2/3	1/4
Na2	2*b*	0.356	0	0	1/4
Mn	2*a*	1.0	0.0	0.0	1/2
O1	4*f*	1.0	2/3	1/3	0.0862(6)

Space group: $P6_3/m\ m\ c$ (194), $a = b = 2.8703$ Å, $c = 11.1533$ Å, $\alpha = \beta = 90°$, $\gamma = 120°$, Vol = 79.577 Å^3, $\chi^2 = 1.7982$. *g*: occupancy. *x*, *y*, and *z*: atomic coordinate.

Supplementary Table 7. Extracted parameters from EXAFS spectra fitting of $Na_{0.612}K_{0.056}MnO_2$.

Scattering path	CN	R (Å)	σ^2 (Å^2)
Mn-O	6	1.889	0.00671
Mn-Mn	6	2.889	0.01057

R-value: 0.00998, S_0^2: 0.80 , E_0: -5.5 eV.

Supplementary Table 8. Extracted parameters from EXAFS spectra fitting of $Na_{0.076}MnO_2$.

Scattering path	CN	R (Å)	σ^2 (Å^2)
Mn-O	6	1.895	0.00516
Mn-Mn	6	2.897	0.00892

R-value: 0.00705, S_0^2 : 0.80 , E_0: -4.1 eV.

Supplementary Table 9. Structure parameters of $K_{0.67}MnO_2$ determined from the XRD Rietveld refinement.

Atom	Site	*g*	*x*	*y*	*z*
K1	8*g*	0.184(5)	-0.196(8)	0.193(9)	1/4
K2	8*g*	0.353(3)	0.261(3)	0.495(2)	1/4
Mn	4*a*	1.0	0.0	0.0	0.0
O1	16*h*	1.0	0.369(1)	0.157(1)	0.007(8)

Space group: *ccmm* (63), a = 5.165(6), b = 2.845(3), c = 12.755(6) Å, $\alpha = \beta = \gamma = 90°$, Vol = 187.525 Å^3 ($Z$ = 4), R_p = 5.51%, R_{wp} = 3.94%, χ^2 = 1.35. *g*: occupancy. *x*, *y*, and *z*: atomic coordinate.

Supplementary Table 10. Structure parameters of $Na_{0.114}K_{0.056}MnO_2$ determined from the XRD Rietveld refinement.

Atom	Site	*g*	*x*	*y*	*z*
Na1	2*d*	0.090(3)	1/3	2/3	1/4
Na2	2*b*	0.024(5)	0	0	1/4
K1	2*d*	0.056(4)	1/3	2/3	1/4
Mn	2*a*	1.0	0.0	0.0	1/2
O1	4*f*	1.0	2/3	1/3	0.069(2)

Space group: $P6_3/m\ m\ c$ (194), $a = b = 2.8465(9)$, $c = 11.1987(2)$ Å, $\alpha = \beta = 90°$, $\gamma = 120°$, Vol = 78.587 Å^3, $R_p = 2.35\%$, $R_{wp} = 3.14\%$, $\chi^2 = 1.265$. *g*: occupancy. *x*, *y*, and *z*: atomic coordinate.

Supplementary Table 11. Structure parameters of $Na_{1.015}K_{0.056}MnO_2$ determined from the XRD Rietveld refinement.

Atom	Site	*g*	*x*	*y*	*z*
Na1	4*c*	0.381(6)	0	-0.0866(3)	1/4
Na2	4*c*	0.633(4)	0	0.6253(4)	1/4
K1	4*c*	0.056(1)	0	0.6253(4)	1/4
Mn	4*a*	1.0	0.0	0.0	0.0
O1	8*f*	1.0	0	0.6438(5)	0.9032(4)

Space group: *cmcm* (63), a = 2.8841(2), b = 5.4855(5), c = 10.8045(2) Å, $\alpha = \beta = \gamma$ = 90°, Vol = 170.938 Å^3 (Z = 4), R_{wp} = 4.44%, χ^2 = 3.58. g: occupancy. x, y and z: atomic coordinate.

Supplementary Table 12. Mixed P2 and OP4 phases of $Na_{0.327}MnO_2$ determined from the XRD Rietveld refinement.

P2- $Na_{0.327}MnO_2$:

Atom	Site	*g*	*x*	*y*	*z*
Na1	2*c*	0.268(6)	1/3	2/3	1/4
Na2	2*b*	0.088(8)	0	0	1/4
Mn	2*a*	1.0	0.0	0.0	1/2
O1	4*f*	1.0	2/3	1/3	0.068(1)

Space group: $P6_3/m\ m\ c$ (194), $a = b = 2.8421(5)$, $c = 11.1947(9)$ Å, $\alpha = \beta = 90°$, $\gamma = 120°$, Vol = 78.266 Å^3. *g*: occupancy. *x*, *y*, and *z*: atomic coordinate.

OP4- $Na_{0.327}MnO_2$:

Atom	Site	*g*	*x*	*y*	*z*
Na1	2d	0.160(5)	2/3	1/3	1/4
Na2	2*c*	0.060(4)	1/3	2/3	1/4
Na3	2a	0.111(2)	0	0	1/2
Mn	4*f*	1.0	2/3	1/3	0.384(4)
O1	4*f*	1.0	1/3	2/3	0.434(6)
O2	8*e*	1.0	0	0	0.341(2)

Space group: $P6_3mc$ (186), $a = b = 2.817(11)$, $c = 17.812(14)$ Å, $\alpha = \beta = 90°$, $\gamma = 120°$, Vol = 122.402 Å^3. *g*: occupancy. *x*, *y* and *z*: atomic coordinate.

Ratio of P2 and OP4: 87.0% : 13.0%, $R_p = 2.60\%$, $R_{wp} = 3.38\%$, $\chi^2 = 3.796$.

Supplementary Table 13. Structure parameters of $Na_{0.998}MnO_2$ determined from the XRD Rietveld refinement.

Atom	Site	*g*	*x*	*y*	*z*
Na1	4*c*	0.363(4)	0	-0.084(8)	1/4
Na2	4*c*	0.640(5)	0	0.641(5)	1/4
Mn	4*a*	1.0	0.0	0.0	0.0
O1	8*f*	1.0	0	0.632(2)	0.900(1)

Space group: *cmcm* (63), a = 2.915(2), b = 5.544(4), c = 10.915(9) Å, $\alpha = \beta = \gamma = 90°$, Vol = 176.434 $Å^3$ (Z = 4), R_p = 2.31%, R_{wp} = 3.69%, χ^2 = 9.454. *g*: occupancy. *x*, *y* and *z*: atomic coordinate.

Supplementary Table 14. Crystal orbital Hamilton populations (COHP) of $Na_{0.555}MnO_2$ and $Na_{0.500}K_{0.055}MnO_2$.

Atoms in $Na_{0.555}MnO_2$	-COHP (eF)	Atoms in $Na_{0.500}K_{0.055}MnO_2$	-COHP (eF)
Na1-O9	0.273	K1-O9	0.180
Na1-O1	0.239	K1-O1	0.179
Na1-O33	0.304	K1-O33	0.178
Na1-O36	0.255	K1-O36	0.177
Na1-O12	0.218	K1-O12	0.191
Na1-O4	0.317	K1-O4	0.193
**average**	**0.267**	**average**	**0.183**
Mn8-O9	1.535	Mn8-O9	1.549
Mn10-O1	1.176	Mn10-O1	1.234
Mn2-O33	1.387	Mn2-O33	1.467
Mn3-O36	1.411	Mn3-O36	1.507
Mn7-O12	1.361	Mn7-O12	1.450
Mn1-O4	0.649	Mn1-O4	1.217
**average**	**1.253**	**average**	**1.404**

Supplementary Table 15. Crystal orbital Hamilton populations (COHP) of $Na_{0.555}MnO_2$ and $Na_{0.500}K_{0.055}MnO_2$. It shows the influence of K on the adjacent Mn-O bonds (Mn2) and distant Mn-O bonds (Mn15, Mn10), as well as Mn atoms at different valence states (the valences for Mn10 and Mn15 are 4+ and 3+, respectively.).

Atoms in $Na_{0.555}MnO_2$	-COHP (eF)	Atoms in $Na_{0.500}K_{0.055}MnO_2$	-COHP (eF)
Mn10-O19	0.709	Mn10-O19	1.399
Mn10-O21	1.282	Mn10-O21	1.494
Mn10-O25	1.340	Mn10-O25	1.491
Mn10-O31	1.442	Mn10-O31	1.283
Mn10-O35	1.490	Mn10-O35	1.456
Mn10-O37	0.790	Mn10-O37	1.545
**average**	**1.176**	**average**	**1.445**
Mn15-O26 (*z* ligand)	1.335	Mn15-O26	1.348
Mn15-O28 (*xy*-plane)	0.848	Mn15-O28	1.372
Mn15-O30 (*xy*-plane)	1.232	Mn15-O30	1.600
Mn15-O48 (*xy*-plane)	1.312	Mn15-O48	1.350
Mn15-O50 (*xy*-plane)	0.649	Mn15-O50	1.429
Mn15-O52 (*z* ligand)	1.589	Mn15-O52	1.553
**average**	**1.161**	**average**	**1.442**
Mn2-O22 (*z* ligand)	1.197	Mn2-O22	1.501
Mn2-O24 (*xy*-plane)	0.743	Mn2-O24	1.455
Mn2-O33 (*xy*-plane)	1.387	Mn2-O33	1.467
Mn2-O34 (*xy*-plane)	1.171	Mn2-O34	1.426
Mn2-O36 (*xy*-plane)	0.799	Mn2-O36	1.480
Mn2-O38 (*z* ligand)	1.579	Mn2-O38	1.579
**average**	**1.146**	**average**	**1.485**

Supplementary Table 16. Crystal orbital Hamilton populations (COHP) of $Na_{0.555}MnO_2$ and $Na_{0.500}K_{0.055}MnO_2$ at their fully charged state.

Atoms in $Na_{0.222}MnO_2$	-COHP (eF)	Atoms in $Na_{0.167}K_{0.055}MnO_2$	-COHP (eF)
Na1-O26	0.356	K1-O19	0.210
Na1-O28	0.330	K1-O22	0.210
Na1-O42	0.329	K1-O23	0.203
Na1-O52	0.356	K1-O26	0.203
Na1-O66	0.340	K1-O35	0.204
Na1-O68	0.339	K1-O38	0.205
**average**	**0.342**	**average**	**0.206**
Mn6-O26	1.511	Mn4-O19	1.618
Mn4-O28	1.639	Mn3-O22	1.619
Mn22-O42	1.554	Mn4-O23	1.504
Mn4-O52	1.558	Mn5-O26	1.547
Mn14-O66	1.575	Mn12-O35	1.373
Mn20-O68	1.513	Mn17-O38	1.548
**average**	**1.558**	**average**	**1.535**

Supplementary Table 17. Crystal orbital Hamilton populations (COHP) of $Na_{0.555}MnO_2$ and $Na_{0.500}K_{0.055}MnO_2$ at their fully discharged state.

Atoms in $NaMnO_2$	-COHP (eF)	Atoms in $NaK_{0.055}MnO_2$	-COHP (eF)
Na1-O35	0.275	K1-O34	0.159
Na1-O39	0.275	K1-O35	0.163
Na1-O43	0.274	K1-O38	0.160
Na1-O47	0.274	K1-O39	0.163
Na1-O52	0.275	K1-O43	0.167
Na1-O56	0.275	K1-O47	0.166
**average**	**0.275**	**average**	**0.163**
Mn20-O35	1.369	Mn23-O34	1.341
Mn17-O39	0.583	Mn22-O35	1.481
Mn20-O43	1.370	Mn25-O38	1.337
Mn22-O47	1.368	Mn24-O39	1.479
Mn28-O52	0.582	Mn22-O43	1.434
Mn21-O56	1.368	Mn24-O47	1.434
**average**	**1.107**	**average**	**1.418**

Supplementary Table 18. Vacancy formation energy in $Na_{0.555}MnO_2$ and $Na_{0.500}K_{0.055}MnO_2$.

Atoms in $Na_{0.555}MnO_2$	Vacancy formation energy (eV)	Atoms in $Na_{0.500}K_{0.055}MnO_2$	Vacancy formation energy (eV)
Na1 (Na_e)	2.712	Na1 (Na_e)	-1.841
Na2 (Na_f)	2.787	Na2 (Na_f)	-3.111
Na3 (Na_e)	3.775	Na3 (Na_e)	-2.971
Na4 (Na_f)	2.294	Na4 (Na_f)	-2.911
Na5 (Na_e)	2.674	Na5 (Na_e)	-3.152
Na6 (Na_e)	2.618	Na6 (Na_e)	-1.419
Na7 (Na_e)	2.934	Na7 (Na_e)	-1.982
Na8 (Na_f)	2.738	Na8 (Na_f)	-3.388
Na9 (Na_f)	2.162	Na9 (Na_f)	-3.024
Na10 (Na_e)	2.755		
**average Na_e**	**2.911**	**average Na_e**	**-2.273**
**average Na_f**	**2.495**	**average Na_f**	**-3.109**

Supplementary Table 19. Diffusion coefficient (D_{Na^+}) from Supplementary Fig. 39.

	D_{O1}(×10^{-11} cm^2s^{-1})	D_{O2}(×10^{-11} cm^2s^{-1})	D_{O3}(×10^{-11} cm^2s^{-1})	D_{A1}(×10^{-11} cm^2s^{-1})	D_{A2}(×10^{-11} cm^2s^{-1})
$Na_{0.612}K_{0.056}MnO_2$	1.952	1.411	1.465	1.561	1.538
$Na_{0.706}MnO_2$	1.296	0.830	0.801	0.969	0.977

Supplementary Methods

Electron paramagnetic resonance (EPR) spectra were obtained using a Bruker electron paramagnetic resonance spectrometer. $MnCl_2$ and MnO_2 after dilution with NaCl were used as standards of Mn^{2+} and Mn^{4+}. The solubility of Mn^{2+} in the electrolytes was analyzed by ultraviolet (UV) spectrum (SPECORD 2010 plus, analytikjena). For UV measurement, the residual electrolyte on the electrodes was washed with dimethyl carbonate (DMC) in an argon-filled glove box. 0.5 and 1.0 mM manganese(II) bis(trifluoromethanesulfonyl)imide ($Mn(TFSI)_2$) in DMC were used to give the standard Mn^{2+} peak. *In situ* differential electrochemical mass spectrometry (DEMS) measurements were carried out using a homemade cell connected to the equipment from Perkin-Elmer (Clarus 680 and SQ 8S).

Iodometric titration:

1. Preparation of standard sodium thiosulfate ($Na_2S_2O_3$) aqueous solution

0.1 M of $Na_2S_2O_3$ aqueous solution is prepared by dissolving 6.25 g of $Na_2S_2O_3 \cdot 5H_2O$ and 5 g of sodium carbonate (Na_2CO_3) in 250 mL of distilled water. The real concentration of $Na_2S_2O_3$ aqueous solution was determined by internal standard method with $K_2Cr_2O_7$ solution.

2. Titration of oxygen in the as-synthesized material

Firstly, 0.1 g of powder sample was dissolved in HCl diluted solution before the iodometric titration under the protection of nitrogen atmosphere. Then, starch solutions and buffered solution were added and the solution changed to dark blue. $Na_2S_2O_3$ aqueous solution was slowly titrated into the solution till the color disappeared. The oxygen non-stoichiometry was then calculated based on the amount of $Na_2S_2O_3$ aqueous solution.
